# Beyond Parent-Compound Removal: Microbial Transformation Pathways, Physiological Constraints, and Evidence Integration for Environmental Steroid Hormones

**DOI:** 10.3390/microorganisms14102160

**Published:** 2026-09-24

**Authors:** Tianyu Zhang, Xiaolu Liu, Tong Zhou, Zhaoxin Li, Guangming Xiong, Edmund Maser, Le Gao, Qingshuang Wang, Hao Zhang

**Affiliations:** 1 School of Life Science and Technology, Changchun University of Science and Technology, No. 7186 Weixing Road, Changchun 130022, China; zty20240517@hotmail.com (T.Z.); liuxiaolu1234@foxmail.com (X.L.);; 2 Department of Pharmaceutics, School of Pharmaceutical Sciences, Jilin University, No. 1266, Fujin Road, Changchun 130021, China; 3 Institute of Toxicology and Pharmacology for Natural Scientists, University Medical School Schleswig-Holstein, Brunswiker Str. 10, 24105 Kiel, Germany

**Keywords:** steroid hormones, microbial degradation, synthetic steroids, cometabolism, redox niches, community carbon flow, stable-isotope tracing, endocrine risk

## Abstract

Steroid hormones occur widely in wastewater, sludge, sediments, soils, and receiving waters, where exposure at low concentrations can elicit pronounced endocrine effects. Microbial transformation is an important removal process, yet loss of a parent compound may reflect phase partitioning, structural modification, steroid-ring cleavage, or mineralization. For synthetic steroids such as 17α-ethinylestradiol (EE2) and synthetic progestins, structural compatibility with entry enzymes and downstream metabolic modules further constrains transformation depth. This review organizes current knowledge around transformation depth, linking substrate recognition to aerobic and anaerobic ring-cleavage pathways, HIP-related downstream metabolism, physiological function, and community carbon flow. Aerobic 9,10-seco and estrogen 4,5-seco pathways and the denitrifying 2,3-seco pathway are supported by metabolite, enzymatic, and genetic evidence, whereas Fe(III)-reducing, sulfate-reducing, and methanogenic systems mainly document reversible redox and stereochemical transformations of natural estrogens. We further distinguish growth-linked utilization, cometabolic modification, and potential detoxification-related homeostatic responses, and examine how community functional organization may extend pathway continuity. Finally, evidence is integrated across metabolic potential, active transformation, and steroid-derived carbon flow, while endocrine-risk outcomes are treated as a parallel environmental endpoint. This framework emphasizes that parent-compound removal alone does not establish degradation depth or risk reduction.

## 1. Introduction

Steroid hormones are signaling molecules characterized by a conserved four-ring scaffold and high biological potency. Natural estrogens, androgens, and progestogens enter the environment primarily through human and livestock excretion, whereas pharmaceutical use and associated waste streams introduce structurally modified steroids such as 17α-ethinylestradiol (EE2) and synthetic progestins. Steroid hormones have been detected in municipal wastewater, sewage sludge, manure-amended soils, surface waters, groundwater, and sediments. Frequently reported compounds include estrone (E1), 17β-estradiol (E2), estriol (E3), testosterone, progesterone, and EE2 [1,2,3,4,5,6,7]. The transformation behavior of diverse natural and synthetic steroids has also been systematically compared in activated sludge [8].

Early studies demonstrated substantial estrogenic activity in wastewater-treatment-plant effluents [9], and effect-directed analyses subsequently identified E1, E2, and EE2 as major contributors to estrogenic fractions [10]. Monitoring studies further showed that these compounds can persist in treated effluents at ng L^−1^ concentrations [2,11]. In vivo studies have linked estrogenic wastewater exposure and EE2 to reproductive and endocrine responses in fish [12,13]. In a whole-lake experiment, chronic exposure of fathead minnows to approximately 5–6 ng L^−1^ EE2 over three open-water seasons caused male feminization, gonadal abnormalities, and ultimately population collapse [14]. Thus, the environmental significance of steroid hormones is determined primarily by their biological potency rather than by their quantitative contribution to bulk organic carbon.

A decline in aqueous parent-compound concentration does not necessarily indicate microbial degradation. Steroids can partition to colloidal organic matter, suspended particles, sludge, soils, and sediments, with the extent of partitioning influenced by hydrophobicity, solid-phase organic carbon, particle characteristics, and matrix composition [15,16,17,18,19,20]. In parallel, microorganisms can catalyze oxidation–reduction, hydroxylation, isomerization, and side-chain remodeling reactions [21,22,23,24,25]. Consequently, aqueous parent loss may reflect phase partitioning, skeleton-retaining biotransformation, steroid-ring cleavage, or mineralization [15,16,17,18,19,20,21,22,23,24,25,26]. These processes differ substantially in environmental consequence because transformation products may retain different persistence, mobility, receptor activity, and carbon fate.

Pure-culture, biochemical, genetic, and omics studies have progressively resolved substrate uptake, entry reactions, and initial ring-cleavage mechanisms for several natural steroids. Some substrates converge, after substrate-specific structural remodeling, on relatively conserved downstream modules associated with the bicyclic intermediate 3aα-H-4α(3′-propanoate)-7aβ-methylhexahydro-1,5-indanedione (HIP) [27,28,29,30]. Aerobic 9,10-seco and estrogen 4,5-seco pathways and the denitrifying 2,3-seco pathway are now supported by comparatively strong metabolite, enzymatic, and genetic evidence [28,30,31,32,33]. By contrast, under Fe(III)-reducing, sulfate-reducing, and methanogenic conditions, current evidence for natural estrogens predominantly describes reversible C17 redox reactions and stereochemical conversion rather than continuous steroid-ring degradation [34]. These differences indicate that both transformation type and transformation depth are strongly dependent on redox context.

An additional challenge is the extrapolation of laboratory pathway studies to environmental conditions. Mechanistic and metabolite-identification experiments often employ steroid concentrations in the high-micromolar to millimolar range to obtain sufficient pathway induction and analytical signal [25,28,29,30], whereas environmental concentrations are commonly in the ng L^−1^ to low μg L^−1^ range [2,3,4,6]. Coexisting organic matter may support growth, enzyme induction, or reducing-equivalent supply, but may also alter steroid transformation through preferential substrate utilization and catabolic regulation [35,36]. Connecting pathway phenotypes established under laboratory conditions with environmentally relevant concentrations, co-substrate composition, and actual metabolic flux is therefore essential for assessing environmental function.

These structure–environment interactions are particularly important for synthetic steroids. For EE2, the C17α-ethinyl substituent may alter recognition by enzymes that evolved for natural estrogens and may constrain compatibility with downstream metabolic modules. EE2 transformation has nevertheless been observed in nitrifying systems, activated-sludge and defined mixed cultures, and during metabolism of natural estrogens [35,37,38]. Determining the subsequent fate of artificial substituents and establishing whether initial transformation proceeds to steroid-ring cleavage and steroid-derived carbon processing remain central unresolved questions.

Environmental steroid transformation is also shaped by microscale heterogeneity and microbial community organization. Reaction–diffusion gradients in sludge flocs and biofilms can create adjacent redox microzones that support different reactions within the same aggregate [39,40]. Community succession, functional-potential analyses, and time-resolved metabolite studies further suggest that multiple populations can participate sequentially in steroid transformation [41]. Linking specific steroid intermediates to their producers, recipients, and subsequent carbon fate is therefore necessary to define the metabolic basis of community-level functional partitioning.

This review uses transformation depth as an organizing concept to integrate substrate-specific entry reactions, steroid-ring cleavage, downstream carbon-skeleton processing, and final carbon fate. The environmental significance of a pathway depends not only on the structural reaction it catalyzes but also on respiratory coupling, cofactor availability, substrate transport, pathway expression, and community organization. We therefore distinguish relatively continuous intracellular pathways from distributed community transformations and evaluate evidence at the levels of metabolic potential, active transformation, and steroid-derived carbon flow. By treating chemical transformation depth, physiological function, environmental constraints, community organization, and endocrine effects as related but non-interchangeable dimensions, we address four questions: what transformation occurs, under what conditions can it persist, which community members process steroid-derived carbon and where does that carbon ultimately go, and does transformation actually reduce endocrine risk?

## 2. Microbial Steroid Transformation Pathways and Their Functional Boundaries

Complete microbial steroid catabolism may involve substrate uptake, initial activation, steroid-ring cleavage, downstream carbon-skeleton processing, and eventual entry of products into central metabolism [27,28,29,30]. The continuity among these stages varies substantially among organisms and environmental conditions. Steroid transformation may therefore range from oxidation–reduction, hydroxylation, isomerization, and structural rearrangement to steroid-ring cleavage and deeper carbon utilization. Viewing these reactions along a common transformation-depth continuum allows different pathways to be compared in terms of both chemical depth and physiological significance.

This section compares the major aerobic and anaerobic pathways and then distinguishes growth-linked utilization, cometabolic modification, and potential detoxification-related responses. The major characterized routes for natural steroids and their convergence toward HIP or HIP-related downstream intermediates are summarized in Figure 1 and Table 1.

### 2.1. Aerobic Steroid Degradation: Structural Activation and Relatively Conserved Downstream Metabolism

Aerobic steroid degradation has the most extensively developed enzymatic and genetic framework. Molecular oxygen functions both as the terminal electron acceptor for respiratory energy conservation and as a direct substrate for monooxygenases and dioxygenases involved in sterol side-chain oxidation, steroid-nucleus activation, and ring cleavage. Different steroid classes use substrate-specific entry reactions, after which some natural steroids converge on relatively conserved downstream modules.

#### 2.1.1. Sterol Side-Chain Degradation and the 9,10-Seco Pathway of Sterols and Androgens

Sterols generally require uptake and C17 side-chain processing before entering steroid-nucleus degradation. In several cholesterol-degrading actinobacteria, the Mce4 system mediates sterol uptake, whereas cytochrome P450 enzymes such as CYP125 initiate terminal side-chain oxidation. Subsequent CoA-dependent, β-oxidation-like reactions progressively shorten the side chain to generate C19 intermediates compatible with steroid-core degradation [27,53,54,55]. CYP142 can partially compensate for CYP125 in some mycobacterial backgrounds, although its relative contribution depends on strain, substrate structure, and expression state [56]. These studies establish a functional distinction between sterol-specific upstream processing and subsequent steroid-core cleavage.

C19 intermediates generated from sterols, together with androgens and other 3-ketosteroids that can enter this module directly, are further processed through the 9,10-seco pathway. KstD catalyzes Δ^1^-dehydrogenation at C1–C2 of the A ring, followed by KshAB-mediated C9α-hydroxylation. The resulting 9α-hydroxylated 1,4-diene intermediate is unstable and undergoes C9–C10 bond cleavage to generate 9,10-seco products [31,32,42,57,58,59]. This reaction sequence defines a major point of convergence between sterol-derived C19 intermediates and androgen catabolism.

#### 2.1.2. A Progesterone-Specific Entry Route Converging on the 9,10-Seco Pathway

Progesterone provides another example of a substrate-specific entry reaction connected to a shared downstream pathway. In *Caenibius tardaugens*, progesterone induces the *pdc* gene cluster, which encodes Baeyer–Villiger monooxygenase (BVMO) and luciferase-like monooxygenase (LLM) activities capable of converting C21 progesterone into testosterone acetate and related C19 steroid esters. These products can subsequently enter androgen-like 9,10-seco catabolism [25]. Although the encoded enzymes show partial functional redundancy, their substrate preferences differ, and changes at C21 or within the A ring can alter catalytic efficiency.

Thus, natural progesterone must first undergo side-chain remodeling to generate a C19 structure compatible with the androgen-type downstream pathway. This defined entry mechanism also provides a useful mechanistic reference for evaluating the structural compatibility of synthetic progestins.

#### 2.1.3. Aerobic Estrogen Degradation Through the 4,5-Seco Pathway

Aerobic bacterial degradation of natural estrogens differs fundamentally from the 9,10-seco pathway because the aromatic A ring requires a distinct activation and cleavage strategy. Early culture-based studies established that phylogenetically diverse bacteria can transform E1 and E2 [60,61,62,63,64], whereas subsequent biochemical and genetic studies identified the 4,5-seco pathway as a major aerobic route.

In *Sphingomonas* sp. strain KC8, E2 is first oxidized to E1 by OecA. E1 is subsequently converted to 4-hydroxyestrone, with OecB implicated in this hydroxylation step, and the Fe-dependent extradiol dioxygenase OecC catalyzes C4–C5 cleavage of the aromatic A ring. Genome/transcriptome analysis, recombinant-enzyme experiments, metal characterization, and ^18^O_2_ tracing collectively established the biochemical basis of this pathway [28]. The characteristic pyridinestrone acid detected in these systems is associated with downstream processing of the unstable cleavage product, although its formation may include abiotic recyclization and therefore should not itself be treated as the direct enzymatic ring-cleavage product.

A related but differently organized pathway has been characterized in *Novosphingobium tardaugens* NBRC 16725. The *edc* system links estrogen uptake, E1 hydroxylation, aromatic-ring cleavage, and subsequent processing of the cleavage product, with genetic analyses supporting specific roles for Edc proteins in these steps [24]. Additional *Novosphingobium* strains further illustrate the distribution of related estrogen-catabolic functions [65].

Actinobacterial estrogen degradation is organized differently. The *aed* gene system identified in *Rhodococcus* spp. contains lineage-specific components associated with E2 oxidation, aromatic-A-ring activation, cleavage, and downstream metabolism [43,44,45,66,67]. More recently, the *ean* cluster characterized in *Sphingomonas colocasiae* C3-2 provided another distinct genetic architecture. EanB1B2B3 mediates estrogen hydroxylation, EanC catalyzes 4,5 cleavage, and EanD participates in subsequent decarboxylation. EanA oxidizes E2 to E1, although functional redundancy among dehydrogenases can partially compensate for its loss. An additional EanE1E2 system outside the main *ean* cluster can mediate C9–C10 cleavage of 4-hydroxyestrone. Current evidence therefore supports 4,5-seco catabolism as the principal growth-associated route in this organism while also revealing a supplementary 9,10-seco branch [46].

Together, these studies show that aerobic estrogen degraders share the general chemical logic of aromatic-A-ring activation and cleavage but can differ substantially in gene-cluster organization and downstream processing.

#### 2.1.4. The Relatively Conserved HIP-Related Downstream Module

HIP is an important bicyclic intermediate formed after extensive processing of steroid A and B rings. Detailed genetic and enzymatic characterization of HIP activation and subsequent C/D-ring cleavage has been obtained primarily from aerobic actinobacteria. HIP has also been detected during denitrifying steroid degradation, indicating that chemically distinct upstream pathways can converge at the level of bicyclic steroid-derived intermediates [29,30,51,52].

In *Rhodococcus* and related actinobacterial models, FadD3 activates free HIP to HIP-CoA. HIP-CoA also functions as an important metabolic signal for the KstR2 regulatory system, thereby coupling production of steroid-derived intermediates to expression of downstream catabolic genes [51,52]. Subsequent β-oxidation-like side-chain processing generates 5-OH HIC-CoA; IpdF and IpdC contribute to its conversion toward a proposed CoA-linked intermediate designated HIEC-CoA in the original pathway model. EchA20 then participates in D-ring hydrolysis to produce COCHEA-CoA, whereas IpdAB contributes to C-ring cleavage. Additional thiolytic and oxidative reactions generate progressively smaller CoA-linked products, including a product assigned as MOODA-CoA in the proposed pathway [29]. Gene-deletion studies cause accumulation of HIP or specific CoA esters and block steroid-supported growth, thereby establishing the functional order of HIP activation, D-ring hydrolysis, C-ring cleavage, and subsequent carbon-skeleton processing.

Although FadD3-mediated activation of free HIP is particularly well established in actinobacteria, the chemical form entering this downstream module may vary among taxa. Some Gram-negative bacteria may generate HIP-CoA directly through upstream reactions or use CoA ligases located outside canonical HIP-associated clusters. Thus, HIP-related C/D-ring catabolism appears relatively conserved at the functional level, whereas entry as free HIP or a CoA ester depends on taxonomic and pathway context.

#### 2.1.5. Community Evidence and the Environmental Extrapolation of Aerobic Pathways

Mechanistically resolved community studies of aerobic steroid degradation have focused mainly on natural estrogens. Activated-sludge enrichments, mixed cultures, and defined consortia have shown that E2-to-E1 conversion, subsequent E1 utilization, and deeper transformation can be distributed among different community members [38,68,69]. By contrast, although batch activated-sludge studies demonstrate transformation of multiple androgens and progestogens [8], community-level studies resolving consecutive intermediates, functional partitioning, and steroid-derived carbon allocation remain limited. Detailed sterol-catabolic mechanisms likewise originate mainly from pure cultures.

An E3-enriched consortium provides a recent example of community-level pathway continuity. The consortium transformed E3 in mineral medium, domestic sewage, and activated-sludge microcosms [41]. Time-resolved metabolite profiles showed sequential formation of putative 16α-hydroxyestrone, E1, 4-hydroxyestrone, pyridinestrone acid, and HIP, supporting entry of E3 into a 4,5-seco-related pathway through E1. Parent loss, community growth, and sequential metabolites consistent with deeper transformation were observed concurrently [41]. However, isotope-resolved E3 carbon balances remain necessary to quantify the fraction of steroid-derived carbon entering biomass, CO_2_, and other carbon pools.

Laboratory concentration is another important boundary. High steroid concentrations facilitate pathway induction and metabolite detection but may change community selection, stress responses, and intermediate accumulation. Microautoradiography combined with fluorescence in situ hybridization (MAR-FISH) analysis of activated sludge demonstrated a concentration-dependent response of the E1-transforming community, with cells associated mainly with Alphaproteobacteria acquiring E1-derived label at sub-μg L^−1^ concentrations [70]. Moreover, ^13^C-labeled pyridinestrone acid was detected during incubation with only 3.7 nM E1 [71], demonstrating that entry into the 4,5-seco pathway can occur near environmentally relevant concentrations. Downstream A/B-ring metabolites have also been characterized [72], although the continuity of these later reactions at environmentally relevant concentrations still requires direct verification.

The shared HIP-related downstream module provides a mechanistic basis for convergence among natural-steroid pathways, but whether synthetic steroids can access this module depends on their upstream structural compatibility. Natural-estrogen degraders can cometabolize EE2 in the presence of E1, E2, or E3, whereas EE2 alone generally does not induce or sustain the same growth phenotype; an E3-enriched consortium likewise showed no substantial EE2 removal [35,41]. These observations highlight entry recognition and the subsequent fate of artificial substituents as major determinants of synthetic-steroid transformation depth.

### 2.2. Anaerobic Steroid Transformation: Independent Entry Reactions and Oxygen-Independent Ring Cleavage

In the absence of molecular oxygen, steroid degradation requires activation and ring-cleavage chemistry distinct from aerobic oxygenase-dependent pathways. Early denitrifying cultures yielded bacteria capable of utilizing cholesterol, estrogens, and androgens and demonstrated that selected steroids can support growth and mineralization during nitrate reduction [73,74,75,76]. Among anaerobic pathways, the denitrifying androgen 2,3-seco route currently has the strongest mechanistic support.

#### 2.2.1. Oxygen-Independent Androgen Degradation Through the 2,3-Seco Pathway

*Steroidobacter denitrificans* and *Sterolibacterium denitrificans* can utilize C19 androgens under nitrate-reducing conditions. Early metabolite studies showed that testosterone is first converted to 1-dehydrotestosterone and then to 1-testosterone before entering oxygen-independent A-ring activation and 2,3-seco cleavage [47,48,77,78,79,80,81].

Studies combining [2,3,4-^13^C]testosterone metabolomics, NMR, and H_2_^18^O tracing established a chemical sequence linking 1-testosterone to a cyclic 1,3-dicarbonyl intermediate and subsequent 2,3-seco products [47]. In *Steroidobacter denitrificans*, AtcABC was biochemically characterized as a bifunctional 1-testosterone hydratase/dehydrogenase. It sequentially hydrates the C1–C2 double bond and oxidizes the resulting C1 hydroxyl group, generating the cyclic 1,3-dicarbonyl intermediate required for subsequent A-ring hydrolysis [82].

Steroid-ring A hydrolase (SRAH) was subsequently identified in *Sterolibacterium denitrificans* and shown directly to hydrolyze the C2–C3 bond of the 17-keto cyclic β-diketone androsta-1,3,17-trione [49]. This finding establishes β-diketone hydrolysis as a key ring-opening reaction of the 2,3-seco pathway. Importantly, direct biochemical evidence for AtcABC and SRAH was obtained from different organisms and substrate systems with different C17 oxidation states. They should therefore be regarded as complementary mechanistic evidence for the oxygen-independent pathway rather than as a single enzymatic sequence fully reconstituted in one organism.

Beyond reaction chemistry, [4-^14^C]testosterone-derived label was recovered in both CO_2_ and biomass during denitrifying growth [83]. This provides direct evidence that androgen-derived carbon is incorporated into cellular material and partially mineralized, thereby linking steroid-ring cleavage to carbon utilization. It does not, however, constitute a completely closed carbon–electron balance.

#### 2.2.2. Cobalamin-Dependent Methylation as an Entry Route for Natural Estrogens

The aromatic A ring of natural estrogens is not directly compatible with the anaerobic androgen 2,3-seco pathway. In *Denitratisoma* sp. strain DHT3, E1 and E2 are first methylated at C10 and converted into C19 androgen-type intermediates, thereby establishing a structural connection between natural estrogens and the anaerobic androgen-catabolic module [84].

The *emtABCD* gene cluster is strongly induced during E2 growth in strain DHT3. Disruption of *emtA* abolishes E2 utilization and estrogen-to-androgen conversion while leaving testosterone utilization intact, demonstrating that the system specifically participates in the estrogen entry reaction [30]. Time-resolved metabolites further show formation of androgen intermediates, 2,3-SAOA, and HIP, supporting subsequent entry into the 2,3-seco pathway.

Cell-extract experiments with *Denitratisoma oestradiolicum* subsequently established S-adenosyl-L-methionine (SAM) as the stoichiometric methyl donor for cobalamin-dependent estrogen methylation. Methyl-^13^C tracing further demonstrated incorporation of the SAM-derived methyl group into androgen products [50]. Thus, DHT3 provides the principal genetic and pathway evidence for the estrogen-specific entry reaction, whereas *D. oestradiolicum* provides direct biochemical evidence for the methyl donor and its regeneration. Maintaining this distinction is important when evaluating the strength of evidence for individual mechanistic steps.

#### 2.2.3. Skeleton-Retaining Transformations Under Other Anaerobic Electron-Accepting Conditions

In lake-sediment and anaerobic-sludge incubations, E2 was oxidized to E1 under nitrate-reducing, Fe(III)-reducing, sulfate-reducing, and methanogenic conditions, and reversible interconversion between E1 and E2 was observed in some systems [85]. 17α-E2 was also detected under Fe(III)-reducing, sulfate-reducing, and methanogenic conditions, consistent with stereoselective reduction through E1.

Unlike the denitrifying 2,3-seco pathway, the major products recovered from these incubations retained the steroid nucleus, and neither defined ring-cleavage products nor direct mineralization of steroid-derived carbon was established. These results are therefore best interpreted as C17 redox and stereochemical transformations rather than continuous degradation of the steroid skeleton [85].

#### 2.2.4. Long-Term Behavior of EE2 Under Anaerobic Conditions

Czajka and Londry examined approximately 5 mg L^−1^ EE2 under nitrate-reducing, Fe(III)-reducing, sulfate-reducing, and methanogenic conditions for 271–725 d, with additional analyses after 35–38 months [86]. No significant biological transformation was detected, despite continued anaerobic metabolic activity in the incubation systems.

These results indicate that EE2 was highly stable in the tested anaerobic communities and culture conditions. They should not, however, be generalized as evidence that EE2 cannot undergo anaerobic transformation under any condition. The molecular basis limiting transformation in these systems remains unresolved.

### 2.3. Structural Compatibility of Synthetic Steroids and EE2 Transformation Modes

Artificial ethynyl groups, halogens, additional methyl groups, altered double bonds, and modified side chains can change the compatibility of synthetic steroids with both entry enzymes and downstream metabolic modules. EE2 loss has been observed during nitrifying cometabolism, natural-estrogen-driven transformation, and heterotrophic bacterial transformation, and several studies have associated these phenotypes with candidate metabolites or functional proteins. The major unresolved question is how artificial substituents influence entry reactions, product branching, steroid-ring cleavage, and eventual steroid-derived carbon fate. Representative structural differences between natural steroids and synthetic analogues, together with the microbial physiological contexts in which EE2 transformation has been reported, are summarized in Figure 2.

#### 2.3.1. Effects of Artificial Substituents on Entry and Downstream Compatibility

Structural modification can influence steroid transformation at two levels. First, an artificial substituent may alter recognition and binding by enzymes evolved for natural steroids. Second, even when an initial reaction occurs, the resulting modified intermediate must remain compatible with subsequent ring-cleavage and carbon-processing modules. Small structural differences between a natural steroid and its synthetic analogue can therefore produce markedly different outcomes, ranging from growth-linked utilization to slow cometabolism, limited structural transformation, or high environmental persistence.

The C17α-ethinyl group of EE2 is a representative example. In *Nitrosomonas europaea*, reported products include modifications involving the ethynyl moiety and introduction of carboxyl functionality, with product profiles varying among physiological and cultivation conditions [87]. Defined ammonia-oxidizer–heterotroph systems further show that different primary EE2 products exhibit distinct downstream stabilities [88]. Earlier heterotrophic studies identified EE2 transformation by *Sphingobacterium* sp. JCR5 and proposed candidate pathways from metabolite profiles, whereas subsequent activated-sludge isolates demonstrated that heterotrophic EE2 transformation is not restricted to a single phylogenetic lineage [89,90].

Synthetic progestins show a similar structural effect. Norgestrel-transforming bacteria isolated from activated sludge formed 4,5-dihydro-norgestrel, 3α,5β-tetrahydro-norgestrel, 6,7-dehydro-norgestrel, and an additional unidentified product [91]. These reactions largely involve reduction or dehydrogenation while retaining the steroid nucleus. Norgestrel was transformed more slowly than natural progesterone, suggesting that artificial structural features affect both reaction efficiency and compatibility with subsequent ring-cleavage pathways.

Comparative activated-sludge experiments have likewise shown large differences in apparent half-lives among natural and synthetic progestogens, glucocorticoids, and other steroid classes [8]. Such studies are useful for identifying persistent structures and selecting compounds for mechanistic analysis, but parent-compound kinetics alone do not resolve entry chemistry, ring cleavage, substituent fate, or final carbon fate.

#### 2.3.2. Nitrifying Cometabolism, Natural-Estrogen-Driven Transformation, and Heterotrophic Evidence for EE2

EE2 transformation has been reported in nitrifying sludge, pure heterotrophic cultures, defined mixed cultures, and environmental microcosms. These systems reveal distinct contributions from nitrifying physiology, substrate induction, candidate functional proteins, and environmental adaptation.

Nitrifying sludge with high ammonia-oxidation activity and cultures dominated by ammonia-oxidizing bacteria (AOB) can transform EE2, whereas systems with weak or inhibited nitrification generally show reduced transformation. Supplemental reducing power may further enhance transformation. Nitrifier enrichments and membrane bioreactors demonstrate that this phenotype can persist under stable nitrifying conditions and at comparatively low EE2 concentrations [37,92,93]. Collectively, these findings support a cometabolic mode maintained by ammonia-oxidizing physiology.

Whole-cell experiments with *N. europaea*, together with AMO homology modeling, molecular docking, and molecular-dynamics simulations, provide additional support for involvement of ammonia monooxygenase (AMO) in EE2 cometabolism [94]. However, because active AMO has not been directly reconstituted as a purified enzyme in this context, this evidence should not be treated as equivalent to direct purified-enzyme catalysis. Moreover, reactive nitrogen species generated during nitrification can contribute to non-biological EE2 transformation [88,95]. EE2 loss in AOB systems must therefore be interpreted as the combined outcome of AMO-associated enzymatic processes, nitrification-associated abiotic chemistry, and, in mixed systems, subsequent heterotrophic transformation.

Natural-estrogen degraders provide a distinct cometabolic mode. Isolates capable of utilizing E1, E2, or E3 transformed EE2 only when natural estrogens were present, including at initial EE2 concentrations of approximately 500–600 ng L^−1^ [35]. EE2 alone did not induce an equivalent physiological response. These observations indicate that enzymes or broader physiological states induced by natural estrogens can extend to a structurally related synthetic substrate.

Heterotrophic pure-culture studies provide additional mechanistic candidates. In *Pseudomonas citronellolis* SJTE-3, SDR3, YjcH, and CYP2 were associated with EE2 transformation, and the strain also removed substantial amounts of EE2 in water, soil, and manure microcosms [96,97]. Nevertheless, the continuous relationship between the reported intermediates, steroid-ring cleavage, and EE2-derived carbon assimilation or mineralization has not been established.

Similarly, *Aeromonas salmonicida* MLN-TP7 showed high apparent EE2 removal and several candidate products, including E1 and lactone-, organic-acid-, and TCA-cycle-like compounds [98]. Because appreciable abiotic loss occurred, biomass increase was not clearly demonstrated, methanol may have contributed to the carbon background, and the proposed downstream products were not connected to EE2 by isotope continuity, these findings support biotransformation but do not directly demonstrate entry of EE2-derived carbon into central metabolism or complete mineralization.

Overall, EE2 can undergo initial transformation under several microbial physiological regimes. However, parent loss may include enzymatic cometabolism, reactive-nitrogen-mediated chemistry, or heterotrophic conversion supported by auxiliary substrates. Transformation depth therefore depends on the identity and stability of primary products and on whether those products can progress to steroid-ring cleavage and subsequent carbon utilization. Representative microbial transformation systems for synthetic steroids, including their physiological contexts, major observations, transformation depth, and current evidence boundaries, are summarized in Table 2.

### 2.4. Growth-Linked Utilization, Cometabolic Modification, and Potential Detoxification-Related Responses

Microbial steroid transformation can be interpreted through three complementary physiological modes: growth-linked utilization, cometabolic modification, and potential detoxification-related responses. These modes respectively emphasize the contribution of a steroid to biomass and energy generation, dependence of transformation on another primary growth substrate, and the role of intermediate removal in maintaining cellular homeostasis. The dominant physiological mode can vary with steroid concentration, electron acceptor, co-substrate availability, and cellular state.

#### 2.4.1. Growth-Linked Utilization

Growth-linked utilization connects steroid uptake, activation, ring cleavage, and downstream carbon processing with cellular proliferation or energy metabolism. The denitrifying estrogen degrader *Denitratisoma* sp. strain DHT3 grows with E2 and nitrate and continuously channels estrogen through the methylation entry reaction into the 2,3-seco pathway [30].

More direct evidence comes from steroid-derived carbon tracing. During denitrifying growth of *Sterolibacterium denitrificans*, [4-^14^C]testosterone-derived label entered both biomass and ^14^CO_2_, directly linking parent consumption with assimilation and partial mineralization of steroid-derived carbon [48]. Under aerobic conditions, *Sphingomonas* sp. strain KC8 can use E1, E2, and testosterone as carbon and energy sources, providing pure-culture evidence for growth-associated natural-estrogen utilization [63,99].

At the community level, an E3-enriched consortium transformed E1, E2, and E3 and generated sequential metabolites consistent with 4,5-seco processing [41]. These observations support growth-associated community transformation, although isotope-resolved carbon allocation is still required to quantify the actual contribution of E3-derived carbon to biomass and mineralization.

Thus, growth or parent disappearance alone should not be considered definitive evidence of complete steroid-carbon utilization. The strongest interpretation combines substrate-dependent growth with consecutive pathway metabolites and, where possible, direct isotope tracing of steroid-derived carbon.

#### 2.4.2. Cometabolic Modification

Cometabolism occurs when steroid transformation depends on metabolic activity sustained primarily by another substrate or physiological process. EE2 provides the clearest examples. Its transformation is associated with active ammonia oxidation in nitrifying systems and can also depend on the presence of natural estrogens in heterotrophic estrogen degraders [35,37,88,94].

In AOB systems, parent EE2 loss is not exclusively enzymatic because reactive nitrogen species generated during nitrification can also transform EE2 [88,95]. Recent work further supports a relationship between ammonia availability, AMO catalytic state, and EE2 transformation [94], reinforcing the need to distinguish enzyme-mediated cometabolism from parallel abiotic reactions.

Coexisting organic substrates can also have contrasting effects. Readily metabolizable carbon may maintain cellular activity, reducing equivalents, and cofactor regeneration, but it can also alter substrate hierarchy or repress steroid-associated pathways. In flow-through experiments, lactate and acetate decreased the apparent transformation rates of testosterone and E2, indicating that the effect of background organic carbon depends on pathway induction, community structure, and substrate-use priorities [36].

Cometabolism can nevertheless initiate transformations that are subsequently continued by other organisms. In defined AOB–heterotroph systems, AOB-mediated primary EE2 transformation was followed by heterotrophic processing, with a fraction of EE2-derived carbon ultimately recovered as CO_2_ [88]. Thus, a non-growth initial transformation can become part of a deeper community-level carbon-processing sequence.

#### 2.4.3. Potential Detoxification-Related Responses and Cellular Homeostasis

Steroid catabolism may contribute to cellular homeostasis by preventing accumulation of metabolically toxic intermediates. When HIP-related downstream metabolism is disrupted, Δ*ipdAB*, Δ*ipdC*, and Δ*echA20* mutants accumulate steroid-derived CoA esters, show depletion of free CoASH, and exhibit HIP- or cholesterol-dependent growth inhibition [29]. These phenotypes demonstrate a cell-protective consequence of maintaining downstream pathway continuity.

The biochemical basis of this toxicity has been further resolved. Brown et al. showed that interruption of cholesterol catabolism causes accumulation of cholesterol-derived acyl-CoAs, depletion of free CoASH, and perturbation of central metabolism. Accumulated CoA species can also inhibit pantothenate kinase, further interfering with CoA homeostasis [100]. In *Mycobacterium abscessus*, deletion of *hsaD* blocks utilization of cholesterol and 4-androstenedione, and exposure to these steroids additionally inhibits growth of the mutant on acetate, indicating that steroid-dependent toxicity may involve intermediate effects beyond CoA depletion alone [101].

Current evidence therefore supports a physiological role for downstream steroid catabolism in preventing harmful intermediate accumulation and maintaining metabolic homeostasis. Because this mechanism has been demonstrated mainly in cholesterol- and androgen-related systems, it is preferable to describe it as a potential detoxification-related or cell-protective function, rather than to infer that steroid pathways evolved primarily for detoxification.

## 3. Environmental Constraints on Steroid-Pathway Operation and Carbon Fate

Pathways validated in model cultures do not necessarily operate continuously in environmental matrices, because actual metabolic flux is constrained by redox conditions, respiratory energy coupling, cofactor supply, substrate bioavailability, and nutritional context. This section therefore focuses on three questions: how redox microenvironments select pathways that can operate; whether steroid oxidation is directly coupled to specific respiratory processes; and whether cofactor availability, mass transfer, and pathway expression reach the functional thresholds required to sustain continuous carbon flow. Together, these environmental and physiological factors determine whether steroid transformation progresses from initial structural modification to steroid-ring cleavage and downstream carbon flow, as summarized in Figure 3.

Microbial steroid transformation is influenced by the local redox environment, respiratory and cofactor support, substrate bioavailability, and nutritional context. These factors can modulate progression from initial structural transformation to steroid-ring cleavage and downstream carbon flow. Representative functional-group changes shown for the initial transformation stage are schematic and do not denote a single conserved entry reaction. Redox microzones within microbial aggregates may create local conditions that differ from the bulk environment, whereas sorption–desorption, diffusion, co-substrates, reducing equivalents, and pathway regulation further influence substrate access and metabolic flux. Cofactors shown in the figure are representative, and their involvement is pathway dependent.

### 3.1. Selection of Steroid Pathways by Redox Microenvironments

Molecular oxygen is both the terminal electron acceptor for aerobic respiration and a reactant in several oxygen-dependent steroid activation and ring-cleavage reactions. Limited oxygen supply can therefore constrain sterol side-chain oxidation, 3-ketosteroid hydroxylation, and aromatic-estrogen cleavage in addition to reducing respiratory capacity. By contrast, the denitrifying 2,3-seco pathway operates without these oxygenase-dependent reactions [28,32,33,55].

Electron acceptors are spatially heterogeneous in activated-sludge flocs, biofilms, sediments, and soil aggregates. Oxygen diffuses from the bulk phase and is progressively consumed, generating gradients from oxic surfaces toward anoxic or anaerobic interiors. Aggregate size, pore architecture, extracellular polymeric substances, local organic loading, cellular respiration, and liquid-phase mass transfer jointly control oxygen penetration and microzone stability [39,40]. Microelectrode studies confirm that bulk dissolved oxygen and organic loading can substantially alter the size of internal anoxic regions in activated-sludge flocs [102].

Bulk dissolved oxygen or oxidation–reduction potential (ORP) therefore provides only an integrated system-level signal and may differ from conditions experienced by individual functional cells. Because ORP reflects multiple redox couples and matrix components, it should ideally be interpreted together with dissolved oxygen, nitrate, Fe(III), sulfate, and corresponding reduction products [102,103].

Substrate structure and organism-specific respiratory capacity further determine whether a redox condition supports a particular steroid pathway. *Denitratisoma* sp. strain DHT3 grows with E2 under nitrate-reducing conditions, with E2 consumption, nitrate reduction, and biomass production occurring concurrently, whereas alternative electron acceptors do not sustain the same growth phenotype [30]. Thus, redox selection reflects not simply electron-acceptor availability but physiological compatibility among substrate entry, respiratory metabolism, and cellular energy conservation.

Overall, oxygenated conditions primarily support oxygen-dependent entry and ring-cleavage pathways, whereas nitrate reduction can sustain the independent 2,3-seco pathway. Fe(III)-reducing, sulfate-reducing, and methanogenic conditions have so far been associated mainly with natural-estrogen redox and stereochemical conversion [34]. Resolving electron-acceptor gradients together with diagnostic steroid metabolites and functional populations at the aggregate scale will be important for understanding how redox microzones shape environmental steroid-carbon flow.

### 3.2. Coupling Steroid Oxidation to Respiratory Energy Conservation

Sustained anaerobic steroid catabolism requires coupling between steroid oxidation, electron-acceptor reduction, and cellular energy conservation. This coupling is best established for denitrifying androgen and natural-estrogen degraders.

In *Sterolibacterium denitrificans*, testosterone consumption occurs together with nitrate reduction and cell growth, while [4-^14^C]testosterone-derived label enters biomass and CO_2_. Consecutive 2,3-seco intermediates and biochemical evidence further connect substrate utilization, steroid-ring cleavage, and denitrifying physiology [33,47,48]. In *Denitratisoma* sp. strain DHT3, E2 consumption, nitrate reduction, biomass formation, the methylation entry reaction, and subsequent 2,3-seco metabolism are similarly coordinated [30]. These systems demonstrate that selected steroids can act as electron donors and carbon sources during anaerobic growth rather than merely undergoing incidental structural transformation.

More complete carbon–electron balances would nevertheless improve quantitative interpretation. Simultaneous measurement of the steroid, major metabolites, nitrogenous reduction products, biomass, and inorganic carbon would allow carbon and electron partitioning to be compared across substrates and organisms.

Fe(III)-reducing, sulfate-reducing, and methanogenic systems currently present a different picture. E2 can be converted to E1 or 17α-E2 under these conditions [34], but direct coupling between steroid oxidation and Fe(III), sulfate, or methanogenic respiration has not been established. Quantitative linkage between steroid transformation and Fe(II), sulfide, or methane production would be required to demonstrate direct respiratory involvement. Isotopically labeled steroids combined with measurements of labeled inorganic carbon or methane could be particularly informative for methanogenic systems.

Auxiliary electron donors and background organic matter further complicate interpretation. Methanol, ethanol, acetate, lactate, or medium-derived organic compounds can maintain cellular activity, drive electron-acceptor reduction, or provide reducing power for cometabolism. The long-term EE2 incubations of Czajka and Londry showed that anaerobic metabolism can remain active while EE2 remains largely untransformed [34]. Thus, electron-acceptor reduction alone does not establish that the target steroid is contributing electrons or carbon.

Denitrifying systems therefore provide the most mature model linking steroid oxidation, ring cleavage, electron-acceptor reduction, and growth. Other anaerobic conditions demonstrate broader structural-transformation potential but require direct carbon–electron coupling before they can be interpreted as continuous respiratory steroid degradation.

### 3.3. Functional Constraints Imposed by Cofactors, Substrate Bioavailability, and Pathway Expression

Appropriate redox conditions and pathway genes are necessary but not sufficient for continuous steroid metabolism. Flux also depends on cofactor assembly and regeneration, effective delivery of substrate to functional cells, and coordinated pathway expression under the prevailing nutritional conditions.

#### 3.3.1. Cofactor Assembly and Regeneration

Several steroid entry and ring-cleavage reactions depend on specific organic or metal cofactors. Cobalamin-dependent estrogen methylation provides a well-resolved example. The reaction requires not only the *emtABCD* system but also cobalamin uptake, cofactor assembly and reductive reactivation, and continued regeneration of SAM through one-carbon metabolism. Proteins associated with estrogen methylation, cobalamin handling, and SAM regeneration are coordinately induced during E2 growth, indicating physiological integration between substrate-specific entry chemistry and cellular cofactor metabolism [30].

Cell-extract experiments further established SAM as the stoichiometric methyl donor for estrogen methylation. Methionine plus ATP, or a broader regeneration system containing serine, tetrahydrofolate, homocysteine, and ATP, can maintain SAM supply and support continued reaction turnover [50]. Thus, pathway activity depends on the metabolic capacity to regenerate required cofactors as well as on the catalytic proteins themselves.

Other pathways show analogous dependencies. CYP125 is a heme enzyme involved in sterol side-chain oxidation [55], KshAB is a Rieske-type oxygenase system [32], and OecC requires iron for aromatic-ring cleavage [28]. AtcABC has been assigned to the xanthine-oxidase family on the basis of sequence and phylogenetic analyses, suggesting potential involvement of molybdopterin-, flavin-, and iron–sulfur-related cofactors, although its complete cofactor composition has not been directly established [33]. The 17β-hydroxysteroid dehydrogenase 0095 (17β-HSD-0095) from *Microbacterium* sp. MZT7 requires NAD^+^ for E2 oxidation, and its activity is also affected by pH and selected metal ions in vitro [104].

Whether cobalamin, cobalt, SAM, metal centers, or other cofactors become quantitative rate-limiting factors in sludge, sediments, or biofilms remains poorly resolved. Linking environmental cofactor status with pathway expression, diagnostic metabolites, and transformation rates would provide a direct test of their ecological relevance.

#### 3.3.2. Solid–Liquid Partitioning, Mass Transfer, and Substrate Bioavailability

Before a steroid can enter a microbial pathway, it must reach functional cells in a bioavailable form. Total steroid concentration therefore differs from the substrate concentration immediately accessible to microorganisms because steroids partition among water, colloids, suspended particles, sludge, soils, and sediment organic matter [15,16,17,18,19].

Aqueous solubility varies among steroid hormones and may influence their accessibility to microbial cells. Reported water solubilities are approximately 13 mg L^−1^ for the natural estrogens E1, E2, and E3, compared with 4.8 mg L^−1^ for the synthetic estrogen EE2 [105], whereas testosterone can reach approximately 23 mg L^−1^ at 25 °C [106]. These values indicate differences in dissolution potential, although actual bioavailability also depends on environmental concentrations, solid–liquid partitioning, and mass transfer. Lower solubility may restrict substrate supply in highly loaded systems, but intrinsic solubility alone does not determine biodegradation rates. For example, low concentrations of Tween 80 enhanced EE2 removal by P*seudomonas citronellolis* SJTE-3, whereas higher concentrations prolonged the degradation lag phase, potentially through micellar sequestration that reduced substrate accessibility [97]. Thus, effective microbial steroid degradation depends on both physicochemical availability and the metabolic capacity of the degrading microorganisms.

Within sludge flocs and biofilms, substrates must additionally cross liquid films, extracellular polymeric matrices, and pore spaces. Aggregate size, porosity, matrix composition, and local cell density influence diffusion distances and mass-transfer rates [39,40]. Consequently, even when a community contains the complete genetic capacity for steroid catabolism, substrate delivery can constrain pathway induction and metabolic flux.

Solid-phase partitioning can have opposing effects. Local retention may increase residence time and create a slowly desorbing substrate reservoir after external loading decreases. Conversely, strong sorption can lower the freely dissolved concentration and reduce transfer to cell surfaces and uptake systems. The relative importance of these effects depends on sorption reversibility, desorption kinetics, microbial spatial organization, and reaction rate.

Field observations likewise indicate that attenuation of E2 and EE2 in rivers depends on both compound properties and environmental microbial activity [107]. Integrating aqueous and solid-phase mass distributions, desorption behavior, and diagnostic metabolites can therefore distinguish long-term retention from genuine biological transformation. Measurements of freely dissolved concentrations or passive-sampling approaches may further improve comparisons among matrices.

Substrate bioavailability thus provides a mechanistic bridge between environmental concentration and intracellular metabolic flux. Total concentration reflects system loading, whereas the freely accessible fraction and mass-transfer regime more directly determine whether steroid molecules reach functional cells and sustain entry reactions.

#### 3.3.3. Coexisting Organic Matter and Pathway Expression

Background organic matter can alter steroid transformation through pathway induction, energy and reducing-equivalent supply, cofactor regeneration, and carbon-source selection. Natural steroids can induce enzyme systems that also transform structurally related synthetic compounds. For example, several natural-estrogen-degrading isolates transform EE2 only in the presence of E1, E2, or E3 [35].

Readily metabolizable carbon can maintain cellular activity and supply reducing equivalents, which may favor transformation of non-growth substrates at trace concentrations. At the same time, alternative carbon sources can alter substrate hierarchy and transcriptional regulation, causing preferential utilization of more accessible carbon. Addition of lactate and acetate decreased apparent transformation rates of selected androgens and estrogens in flow-through cultures [36]. Background carbon therefore does not necessarily stimulate steroid transformation; its effect is substrate- and system-dependent.

Nutritional conditions also influence pathway expression and final carbon fate. Quantitative proteomic studies have linked background nitrogen and carbon conditions to changes in proteins associated with microbial E1 degradation [108], and ^13^C-E2 sediment microcosms show that nutrient background and community succession can affect E2 mineralization [109]. Proteomic responses to E2 have likewise been characterized in defined estrogen-degrading strains [110]. These observations reinforce the need to consider nutritional context when extrapolating pathway capacity to environmental flux.

## 4. Community-Level Steroid Transformation: Functional Organization and Carbon Flow

Environmental steroid transformation can be organized in different ways. Some microorganisms connect substrate entry, steroid-ring cleavage, and downstream carbon processing within the same cell, forming relatively continuous pathways [29,51,52,53]. In contrast, mixed cultures and enrichment communities can display broader substrate ranges, community-specific products, and sequential parent/intermediate transformation involving multiple members [38,41,68,69]. Studies of E2 biodegradation under aerobic and anaerobic conditions and E1 biodegradation in activated sludge further document changes in microbial community composition during steroid transformation [111,112].

From a functional-organization perspective, steroid-transforming systems can be conceptualized as two complementary modes: relatively continuous intracellular pathways and distributed community transformation (Figure 4). The first emphasizes pathway continuity within individual functional populations, whereas the second emphasizes complementary reactions partitioned among multiple populations. Understanding how steroid-derived carbon is allocated among these organisms, and which exchangeable intermediates connect them, is central to resolving community-level steroid metabolism.

### 4.1. Relatively Complete Pathway Organisms and Distributed Functional Guilds

#### 4.1.1. Relatively Continuous Intracellular Pathways

Some steroid degraders connect uptake, substrate-specific entry, steroid-ring cleavage, and at least part of downstream carbon processing within a single organism. Cholesterol-degrading actinobacteria provide the most thoroughly characterized examples. In *Rhodococcus jostii* RHA1 and mycobacteria, sterol uptake, side-chain processing, A/B-ring cleavage, and HIP-related C/D-ring metabolism can occur sequentially within the same cell [29,51,52,53]. Such organization allows steroid-specific intermediates to be processed predominantly intracellularly and reduces dependence on extracellular release and downstream community partners.

Other bacteria use multiple substrate-specific entry routes connected to partially shared downstream metabolism. *Caenibius tardaugens* NBRC 16725 can transform natural estrogens, C19 androgens, and natural progesterone. These substrates undergo distinct initial structural remodeling before connecting with overlapping downstream steroid-catabolic functions [24,25,113]. Substrate specificity can therefore be concentrated at pathway entry, whereas deeper metabolism becomes increasingly shared.

A comparable organization occurs under denitrifying conditions. *Denitratisoma* sp. strain DHT3 uses *emtABCD*-associated cobalamin-dependent methylation to convert natural estrogens into C19 androgen intermediates and subsequently forms 2,3-SAOA and HIP, thereby connecting the estrogen-specific entry reaction to the 2,3-seco pathway [30]. Coordinated induction of estrogen methylation, cobalamin processing, and SAM-related metabolism further illustrates physiological integration among entry chemistry, cofactor supply, and downstream catabolism [50].

Relatively continuous intracellular organization therefore reflects not merely coexistence of pathway genes but coordination among entry reactions, ring cleavage, downstream carbon processing, cofactor metabolism, and energy conservation.

#### 4.1.2. Distributed Functional Organization: Metabolic Relay and Auxiliary Dependence

Mixed cultures and enrichment communities show that different stages of steroid transformation can also be partitioned among different organisms. In an EE2 system, ammonia oxidizers primarily generate initial products, whereas heterotrophic bacteria subsequently process selected EE2-derived metabolites [88]. In an E3-enriched community, individual isolates mainly perform early transformation, whereas the intact consortium generates a more continuous sequence of 4,5-seco-related products [41].

Defined two-member cultures provide additional phenotypic evidence for functional complementarity. A mixed culture of *Rhodococcus equi* DSSKP-R-001 and *Comamonas testosteroni* QYY20150409 removed more E2 than either monoculture and produced three candidate products not observed in the individual cultures [69]. However, because these products were assigned mainly from mass-spectrometric data and member-specific production and uptake were not determined, this experiment does not yet establish a defined cross-member steroid-intermediate relay.

Community carbon transfer should also be separated into steroid-specific metabolic relay and general downstream carbon flow. Exchange of an intact steroid or a diagnostic steroid-pathway intermediate can directly connect different stages of steroid catabolism. In contrast, low-molecular-weight compounds generated after extensive ring cleavage may enter general community metabolism and be used by secondary consumers that never transform the parent steroid [29,114,115]. Similarly, interspecies transfer of H_2_, formate, or other reducing equivalents [116,117] and sharing of cobamides [118,119] can support community metabolism without constituting direct exchange of steroid intermediates.

This distinction prevents general syntrophy or nutrient exchange from being overinterpreted as evidence for steroid-specific cross-feeding.

### 4.2. Community Attribution of Steroid-Derived Carbon: Current Evidence and Limits

Member-level attribution of steroid-derived carbon remains much less developed than pathway identification. In pure cultures, isotope experiments provide direct attribution because a single organism is responsible for substrate processing. The [4-^14^C]testosterone experiment with *Sterolibacterium denitrificans*, for example, directly showed steroid-derived carbon entering biomass and CO_2_ [48].

In mixed systems, isotope interpretation is more complex. The AOB–heterotroph EE2 system showed that ammonia oxidizers primarily form initial EE2 products, whereas heterotrophic cultures continue transformation and generate ^14^CO_2_. Sequential reactors further demonstrated that 4-hydroxy-EE2 produced during the ammonia-oxidizing stage can be consumed during the subsequent heterotrophic stage [88]. This provides direct evidence that different phases of steroid-derived carbon processing can be distributed among functional groups.

Natural-estrogen communities provide complementary evidence based on temporal association. During E3 transformation, putative 16α-hydroxyestrone, E1, 4-hydroxyestrone, pyridinestrone acid, and HIP appear sequentially while the community composition shifts toward dominance by *Croceicoccus estronivorus* [41]. These observations link metabolic stages to community succession but do not quantitatively assign individual metabolites or carbon fractions to specific members.

MAR-FISH has been used to identify cells acquiring estrogen-derived label in activated sludge. At sub-μg L^−1^ E1 concentrations, most cells incorporating [^3^H]E1-derived label were associated with Alphaproteobacteria [70]. However, isotope incorporation alone cannot determine whether a cell assimilated the parent E1, a steroid-specific intermediate, or a downstream metabolite. Metabolite-resolved temporal information is therefore required.

Functional markers can narrow the range of candidate organisms. For example, lineage-associated *aedJ* and *edcC* markers have been used to detect and quantify potential estrogen degraders in environmental samples [45]. However, gene presence indicates metabolic potential rather than actual steroid-carbon assimilation. Stable-isotope probing and single-cell approaches are required to bridge this gap.

DNA-based stable-isotope probing (DNA-SIP) and RNA-based stable-isotope probing (RNA-SIP) can associate labeled-carbon assimilation with active microbial populations, whereas fluorescence in situ hybridization coupled with nanoscale secondary ion mass spectrometry (FISH-NanoSIMS) can further combine taxonomic identity, spatial context, and isotopic enrichment [118,119,120,121,122]. Although these methods remain underused in environmental steroid studies, combining them with position-specific labeled steroids, time-resolved metabolites, and pathway expression could distinguish direct parent-steroid utilizers, consumers of steroid-specific intermediates, and secondary consumers of shared downstream carbon.

### 4.3. Cross-Member Transformation of Steroid Intermediates: Current Evidence and Structural Constraints

The closest current evidence for sequential cross-member transformation of a steroid-specific substrate comes from the EE2 ammonia-oxidizer–heterotroph system. Ammonia oxidizers form 4-hydroxy-EE2, sulfo-EE2, and nitro derivatives, whereas downstream heterotrophic cultures further transform 4-hydroxy-EE2 and selected nitro products and convert a fraction of EE2-derived carbon to CO_2_ [88]. Different intermediates show different downstream accessibility, with sulfo-EE2 remaining comparatively persistent under the tested conditions.

The E3-enriched consortium provides a second type of evidence for distributed natural-estrogen transformation. Individual isolates predominantly form putative 16α-hydroxyestrone, whereas the intact consortium proceeds to E1, 4-hydroxyestrone, and 4,5-seco-related products [41]. This strongly supports stage-specific functional complementarity, although direct producer–receiver relationships for individual intermediates remain unresolved.

Chemical form and cellular localization are likely to constrain cross-member exchange. Free E1, hydroxylated estrogens, and some ring-cleavage products can occur extracellularly. Pyridinestrone acid, for example, has been detected in extracellular fractions during E1 transformation [71], making physical access by other organisms possible. Extracellular detection, however, does not demonstrate uptake by another member. In contrast, HIP-CoA and downstream CoA esters are predominantly intracellular metabolic forms, and direct evidence for their release and reacquisition across cells is lacking [29,51,52].

Artificial structural modifications can further alter the compatibility of intermediates with downstream members. Different primary EE2 products exhibit distinct capacities for subsequent heterotrophic transformation [88], and several natural-estrogen degraders transform EE2 mainly when E1, E2, or E3 induces an appropriate physiological state [35]. Community continuity therefore depends not only on the presence of multiple functional populations but also on whether an intermediate has the appropriate chemical structure and cellular accessibility for downstream recognition.

Functional stability also depends on long-term selection. The E3-enriched consortium retained its transformation phenotype under estrogen selection but lost E3-degrading capacity after propagation with dimethyl sulfoxide (DMSO) in the absence of estrogen and subsequent reinoculation into E3 medium [41]. Substrate selection pressure is therefore an important determinant of community functional persistence.

Current evidence has thus progressed from single-organism pathways to functional complementarity, sequential transformation, and community succession. The next critical step is to connect structurally defined intermediates with specific producers, recipients, and steroid-derived carbon flux.

## 5. Evidence Integration for Microbial Transformation of Environmental Steroids: From Metabolic Potential to Steroid-Derived Carbon Flow

Different experimental approaches answer different questions about environmental steroid transformation. Genomes, metagenomes, and functional genes identify potential metabolic capacity. Substrate-dependent expression, time-resolved metabolites, enzyme assays, and genetics establish whether specific reactions actually operate. Isotope tracing and mass balances further address assimilation, mineralization, and biological allocation of steroid-derived carbon. Based on these advances, the evidence discussed in this review is organized into three complementary observation dimensions—metabolic potential, active transformation, and steroid-derived carbon flow—while endocrine risk is treated as a parallel environmental endpoint (Table 3). Representative studies discussed in this review, including their experimental contexts, major transformation outcomes, analytical and mechanistic evidence, and corresponding interpretive boundaries, are further summarized in Table S1.

### 5.1. Metabolic Potential: Distribution and Environmental Identification of Known Pathways

Mechanistic studies of natural-steroid catabolism have established genetic markers that can be used to identify potential steroid-transforming capacity. In actinobacteria, sterol uptake and 9,10-seco catabolism involve modules including Mce4-mediated sterol uptake, CYP125-associated side-chain oxidation, and KstD/KshAB-mediated steroid-core activation [27,31,32,53,54,55,56]. Aerobic estrogen 4,5-seco pathways are represented by lineage-associated *oec* [28], *edc* [24], *aed* [43,44,45], and *ean* [46] organizations. Denitrifying androgen degradation involves 2,3-seco-associated modules [33,47,48,49], whereas natural-estrogen entry additionally requires the *emtABCD*-associated cobalamin-dependent methylation system [30]; SAM-dependent methyl transfer and related one-carbon metabolism were subsequently characterized biochemically in *D. oestradiolicum* [50].

These markers provide reaction-specific information. KstD and KshAB are associated with activation of 3-ketosteroids for 9,10-seco degradation, whereas proteins such as OecC, EdcB, AedB, and EanC are associated with cleavage of the aromatic-estrogen A ring. The *emt* system indicates the capacity to remodel natural estrogens into androgen-type intermediates under denitrifying conditions. Interpreting markers as combinations spanning substrate uptake, entry chemistry, ring cleavage, and HIP-related downstream processing is therefore more informative than relying on a single homologous gene.

Comparative genomic analysis has shown that pathway–gene combinations from characterized organisms can be used to identify candidate steroid degraders and compare metabolic capacity among phylogenetic groups [123]. Metagenomic marker analyses based on known 9,10-seco proteins further indicate that bacterial steroid-catabolic potential is widely distributed across engineered, soil, rhizosphere, marine, and host-associated environments [124]. At this level, genes primarily answer the question what capacity may be present. Linking them to substrate-responsive expression, diagnostic metabolites, and transformation phenotypes is required to establish actual pathway operation.

### 5.2. Active Transformation: From Structural Change to Continuous Pathway Operation

Active-transformation evidence directly describes the chemical reactions experienced by a target steroid under a defined condition. Time-resolved metabolites establish reaction sequences, transcript or protein induction associates pathways with substrate response, and purified enzymes or genetic perturbations identify catalytic components. Agreement among these approaches can directly connect parent loss with reaction order, ring-cleavage position, and pathway function.

Skeleton-retaining transformation and steroid-ring cleavage represent different depths of active transformation. Under several anaerobic conditions, E2 is converted to E1 and 17α-E2 while the steroid nucleus remains intact [34]. By contrast, the aerobic estrogen 4,5-seco pathway has direct biochemical evidence for C4–C5 cleavage by OecC [28], with subsequent A/B-ring metabolites characterized in downstream studies [72]. Detection of ^13C-labeled pyridinestrone acid from 3.7 nM ^13C-E1 in activated sludge further demonstrates that the characteristic 4,5-seco entry chemistry can operate near environmentally relevant concentrations [71].

The denitrifying 2,3-seco pathway provides another example of increasingly integrated evidence. [2,3,4-^13C]testosterone metabolites, H_2_^18O tracing, and AtcABC biochemistry established key reactions in androgen A-ring activation [33,47], whereas SRAH characterization directly confirmed C2–C3 hydrolysis of a cyclic β-diketone intermediate [49]. For natural estrogens, time-resolved metabolites, *emtA* disruption, and pathway expression in *Denitratisoma* sp. DHT3 connect estrogen-to-androgen conversion with subsequent 2,3-seco metabolism [30]. Cell-extract studies in *D. oestradiolicum* independently establish SAM as the methyl donor for the entry reaction [50].

Synthetic steroids show more heterogeneous evidence. EE2 can undergo nitrification-associated transformation, natural-estrogen-dependent cometabolism, and heterotrophic transformation, and individual systems have yielded candidate products or functional components [35,37,96,97,98]. In defined AOB–heterotroph systems, selected initial products generated by AOB can be subsequently transformed by heterotrophs [88]. Nevertheless, nitrifying systems also contain reactive-nitrogen-mediated abiotic reactions [88,95], and many heterotrophic candidate pathways still lack continuous evidence connecting artificial-substituent processing to steroid-ring cleavage and final carbon fate.

Active transformation therefore supports conclusions about what reactions actually occur, but the strength of interpretation depends on whether parent loss is linked to defined products, catalytic components, and pathway continuity.

### 5.3. Steroid-Derived Carbon Flow: From Isotope Attribution to Member-Level Allocation

Steroid-derived carbon flow addresses a question that metabolite and gene data alone cannot answer: Where does the carbon originating from the steroid actually go? Direct evidence requires isotope continuity into biomass, inorganic carbon, or other quantitatively defined carbon pools, ideally supported by mass balance.

The denitrifying testosterone system provides one of the clearest examples. [4-^14^C]testosterone-derived carbon was recovered in both biomass and CO_2_ during growth of *Sterolibacterium denitrificans* [48]. Together with 2,3-seco metabolite and pathway evidence, this demonstrates that steroid-ring transformation is connected to assimilation and partial mineralization.

EE2 studies further show that steroid-derived carbon processing can be partitioned among different functional groups. Ammonia oxidizers generate 4-hydroxy-EE2, sulfo-EE2, and other products; some nitro derivatives may additionally form abiotically under high-nitrite conditions. Heterotrophic cultures subsequently transform selected products and convert part of the EE2-derived label into CO_2_, while sequential cultures demonstrate continued removal of AOB-derived 4-hydroxy-EE2 [88,95]. These experiments integrate initial structural modification, downstream transformation, and partial mineralization within one carbon-flow framework.

Natural-estrogen consortia provide a complementary community context. During E3 transformation, sequential formation of putative 16α-hydroxyestrone, E1, 4-hydroxyestrone, pyridinestrone acid, and HIP occurs together with pronounced community succession [41]. Although this associates metabolic stages with candidate functional populations, quantitative member-level attribution of E3-derived carbon remains unresolved.

Stable-isotope approaches provide the methodological basis for resolving this problem. DNA-SIP and RNA-SIP can associate labeled-carbon assimilation with active microbial populations, whereas FISH–NanoSIMS can integrate taxonomic identity, spatial position, and isotope enrichment [120,121,122,125,126]. Combined with strategically positioned isotope labels, time-resolved metabolomics, and pathway-expression data, these methods could distinguish organisms that directly use the parent steroid from those consuming steroid-specific intermediates or downstream shared carbon.

Steroid-derived carbon flow is therefore not simply another form of pathway evidence. It connects chemical transformation to biological fate and provides the basis for determining which members actually assimilate or mineralize carbon originating from the steroid.

### 5.4. Endocrine Risk as a Parallel Endpoint to Transformation Depth

Transformation depth and endocrine risk are related but represent different environmental dimensions. Carbon-flow analysis describes how the steroid structure and its carbon skeleton are transformed, assimilated, or mineralized, whereas endocrine effects depend on the receptor activity and relative abundance of the parent compound and its products. Chemical fate and effect-based measurements should therefore be evaluated in parallel.

Several natural-estrogen studies show that deeper structural transformation can coincide with reduced estrogenic activity. Degradation of E2 by *Rhodococcus zopfii* and *R. equi* was accompanied by a marked decline in estrogenic activity [62]. In the 4,5-seco pathway of *Sphingomonas* sp. strain KC8, yeast estrogen-reporter assays showed negligible estrogenic activity for the characteristic product pyridinestrone acid, while the overall estrogenic activity of the culture decreased as E1 was consumed and pathway intermediates changed [28]. These experiments directly connect defined structural transformations with reduced biological effect.

However, different transformation stages do not contribute equally to risk reduction. Oxidation of E2 to E1 changes the parent structure, but E1 remains estrogenically active [12]. Some hydroxylated or other skeleton-retaining products may likewise retain receptor activity [28]. Steroid-ring cleavage usually represents deeper structural destruction, but net environmental risk still depends on product formation, persistence, disappearance, and residual activity.

Chemical and effect-directed analyses therefore provide complementary information. Time-resolved quantification of parent compounds and major metabolites describes changing mixture composition, whereas receptor-reporter or other biological assays measure net activity at the same time points. Combining these approaches can determine whether risk reduction accompanies parent loss, formation of low-activity products, steroid-ring cleavage, or subsequent depletion of downstream intermediates.

Overall, microbial structural transformation can reduce steroid endocrine activity, but parent disappearance alone does not demonstrate detoxification. Integrating chemical fate with biological-effect measurements is necessary to establish how transformation depth relates to actual risk reduction.

## 6. Conclusions

Microbial transformation of environmental steroids spans a continuum from substrate recognition and uptake to specific entry reactions, steroid-ring cleavage, downstream carbon-skeleton processing, assimilation, and mineralization. Different steroid classes impose distinct upstream structural requirements, but natural sterols, androgens, estrogens, and progestogens can, after appropriate structural remodeling, converge to varying degrees on relatively conserved downstream metabolism. The concept of transformation depth therefore provides a useful framework for linking parent-compound modification with ring destruction, final carbon fate, physiological function, and environmental significance.

Mechanistic understanding of natural-steroid catabolism has advanced substantially. Aerobic 9,10-seco and estrogen 4,5-seco pathways define distinct activation and ring-cleavage mechanisms for 3-ketosteroids and aromatic estrogens [28,31,32], whereas HIP-related studies resolve subsequent processing of the residual C/D-ring skeleton [29]. The denitrifying 2,3-seco pathway establishes an oxygen-independent mechanism for androgen A-ring cleavage, and cobalamin-dependent methylation allows natural estrogens to be structurally remodeled into this pathway. Isotope tracing further demonstrates assimilation and partial mineralization of androgen-derived carbon [30,48]. In contrast, Fe(III)-reducing, sulfate-reducing, and methanogenic systems currently provide stronger evidence for skeleton-retaining redox and stereochemical transformation than for continuous ring degradation [34].

Research on synthetic steroids is progressing from parent-removal kinetics toward structural compatibility and pathway continuity. EE2 can be transformed during nitrification, natural-estrogen-induced metabolism, and heterotrophic bacterial activity [35,37,96,97], and different systems produce distinct candidate product branches [87,88,98]. Synthetic progestin studies similarly indicate that artificial structural modification can alter entry compatibility, reaction rate, and product type [25,91]. Determining the fate of artificial substituents and connecting these transformations to steroid-ring cleavage and steroid-derived carbon flux will be essential for defining the actual environmental fate of synthetic steroids.

Environmental pathway operation is further controlled by microbial physiology and community organization. Electron acceptors, cofactors, substrate bioavailability, and coexisting organic matter regulate pathway expression and metabolic flux, whereas transformation can occur through either relatively continuous intracellular pathways or complementary reactions distributed among multiple community members. The AOB–heterotroph EE2 system demonstrates sequential initial and downstream transformation with partial conversion of EE2-derived carbon to CO_2_ [88], whereas E3-enriched consortia connect sequential metabolites with community succession and substrate-dependent maintenance of the degradation phenotype [41]. Direct producer–receiver relationships for specific steroid intermediates nevertheless remain largely unresolved.

Metabolic potential, active transformation, and steroid-derived carbon flow should therefore be regarded as complementary observation dimensions. Functional genes define what a microbial system may be capable of doing; metabolites, expression, enzymology, and genetics establish which reactions actually occur; and isotope tracing identifies the biological fate of steroid-derived carbon. Endocrine effects constitute a parallel environmental endpoint that determines whether chemical transformation is accompanied by meaningful risk reduction.

Future work should prioritize continuous pathway validation at environmentally relevant concentrations, direct resolution of artificial-substituent fate, member-resolved steroid-carbon allocation, and simultaneous measurement of chemical transformation and biological effects. Integrating pathway biochemistry, environmental physiology, community organization, and isotope-resolved carbon fate will allow environmental steroid research to move beyond descriptions of parent-compound removal toward mechanistic understanding of transformation depth, functional organisms, final carbon fate, and risk reduction.

## Figures and Tables

**Figure 1:**
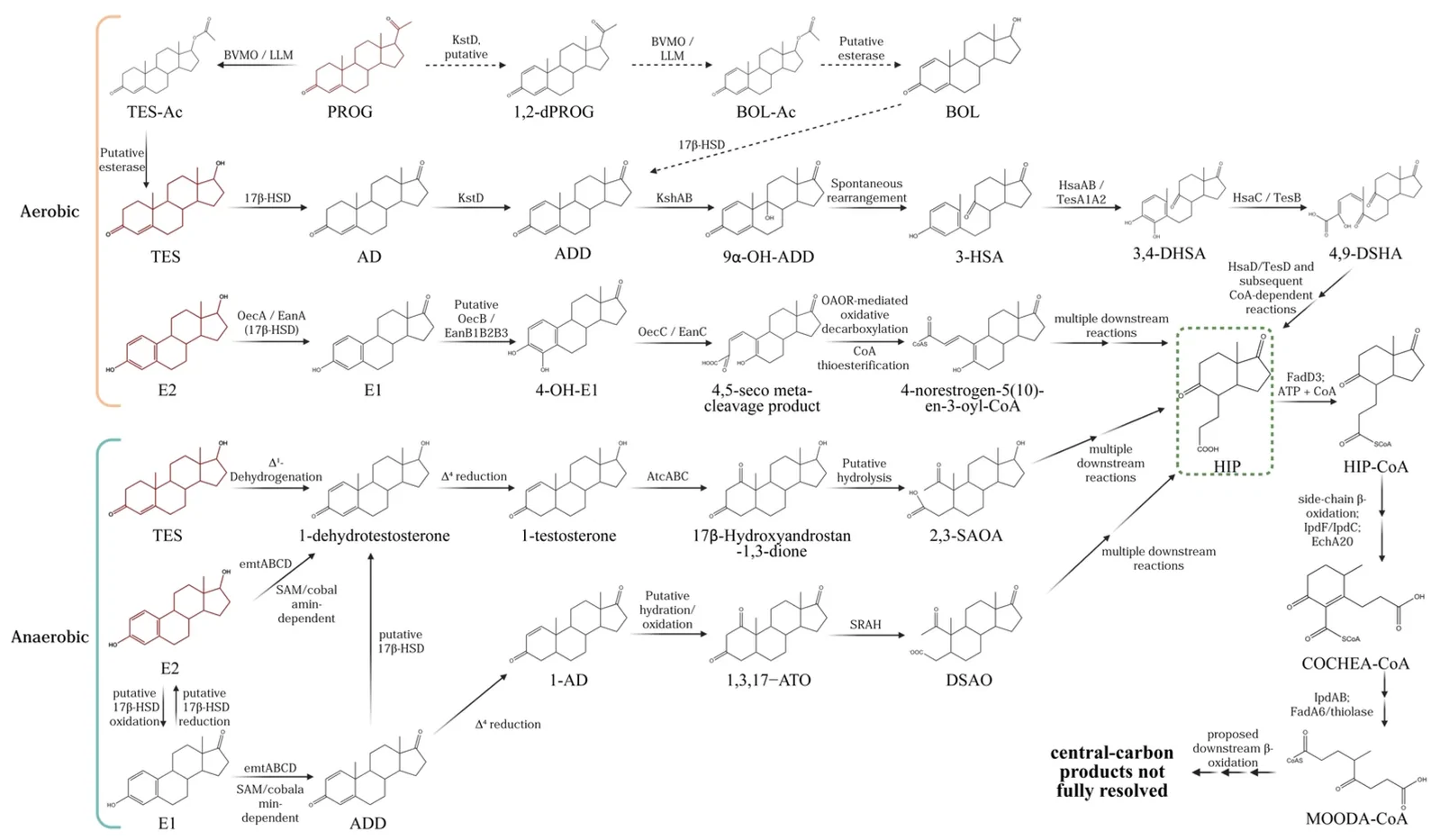
Overview of microbial steroid transformation pathways under aerobic and anaerobic conditions. Representative aerobic and anaerobic microbial catabolic routes for natural steroids and their convergence toward HIP-related downstream metabolism. Aerobic pathways include C21-to-C19 remodeling of progesterone, the 9,10-seco pathway of 3-ketosteroids, the estrogen 4,5-seco pathway, and downstream processing of HIP-related intermediates. Anaerobic pathways include the 2,3-seco route for androgen degradation and the cobalamin- and S-adenosyl-L-methionine (SAM)-dependent conversion of natural estrogens to androgen intermediates before entry into the 2,3-seco pathway. Selected characterized pathways converge at HIP or HIP-related intermediates; subsequent CoA-dependent C/D-ring processing is particularly well characterized in actinobacteria. AtcABC-mediated hydration/oxidation of 1-testosterone (17β-hydroxy substrate) was demonstrated in *Steroidobacter denitrificans*, whereas SRAH-mediated C2–C3 hydrolysis of androsta-1,3,17-trione (17-keto substrate) was demonstrated in *Sterolibacterium denitrificans*; the corresponding reactions on alternative C17 substrates remain putative and do not represent a fully reconstituted pathway in one organism. Within the progesterone-remodeling branch, dashed arrows distinguish an alternative route in which a fraction of progesterone is first converted to 1,2-dehydroprogesterone before proceeding through boldenone-related intermediates; the dashed line style does not denote a difference in experimental evidence strength. Paired opposing arrows denote bidirectional redox interconversion between E2 and E1. Double and triple forward arrows indicate condensed sequences of multiple downstream reactions, with intermediate steps omitted for clarity; the number of arrows does not denote the exact number of omitted reactions. Parent compounds are shown in red. Abbreviations: PROG, progesterone; 1,2-dPROG, 1,2-dehydroprogesterone; TES-Ac, testosterone acetate; BOL-Ac, boldenone acetate; BOL, boldenone; TES, testosterone; AD, androstenedione; ADD, androsta-1,4-diene-3,17-dione; 9α-OH-ADD, 9α-hydroxyandrosta-1,4-diene-3,17-dione; E1, estrone; E2, 17β-estradiol; 4-OH-E1, 4-hydroxyestrone; 2,3-SAOA, 17β-hydroxy-1-oxo-2,3-seco-androstan-3-oic acid; HIP, 3aα-H-4α(3′-propanoate)-7aβ-methylhexahydro-1,5-indanedione; SAM, S-adenosyl-L-methionine; CoA, coenzyme A.

**Figure 2:**
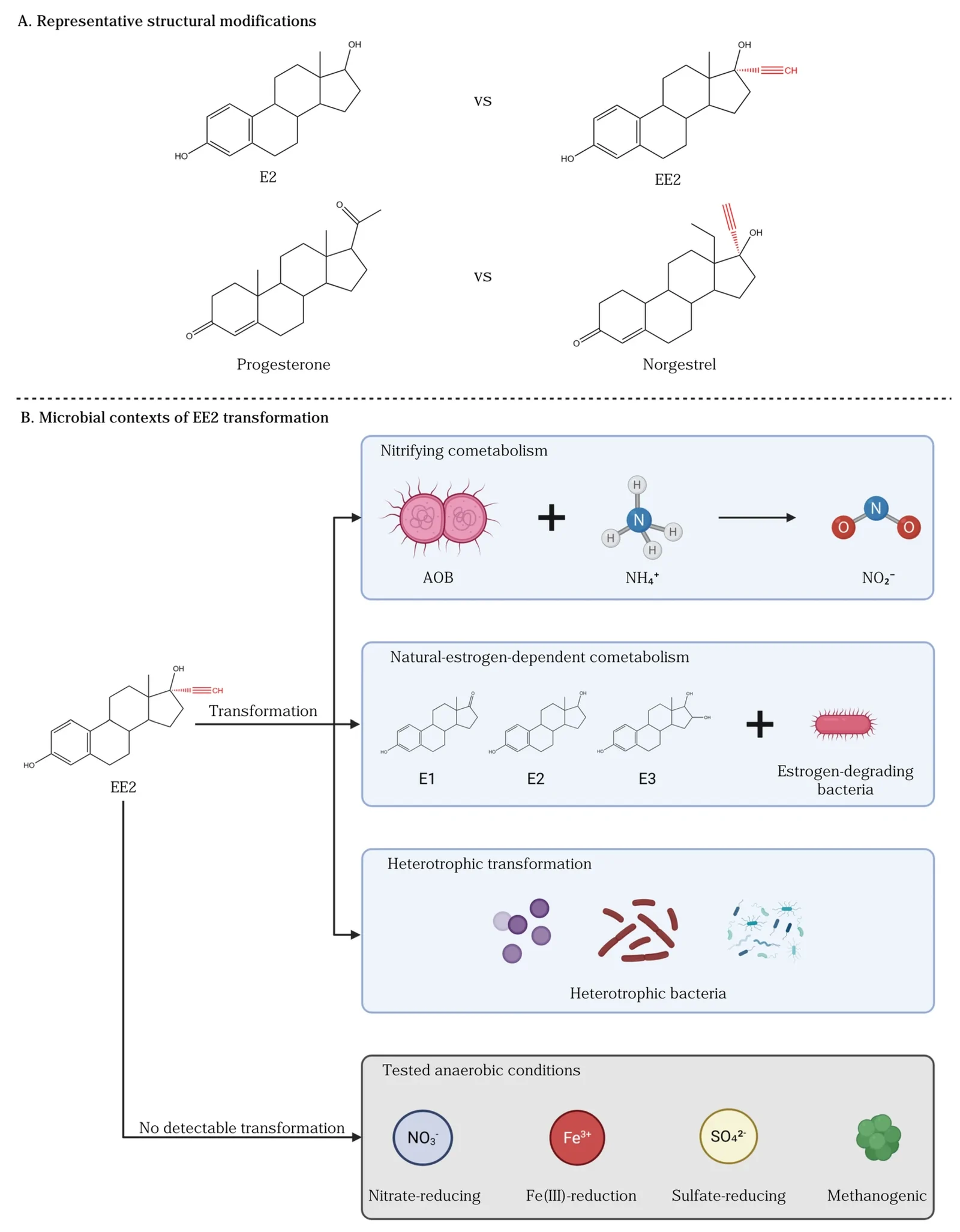
Representative structural modifications of synthetic steroids and microbial contexts associated with EE2 transformation. (**A**) Structural comparison of natural steroids and representative synthetic analogues, illustrated by E2 versus 17α-ethinylestradiol (EE2) and progesterone versus norgestrel; representative artificial substituents are highlighted in red. (**B**) EE2 transformation has been reported under nitrifying cometabolism, natural-estrogen-dependent cometabolism, and heterotrophic bacterial transformation. In contrast, no detectable EE2 biotransformation was observed under the tested long-term nitrate-reducing, Fe(III)-reducing, sulfate-reducing, and methanogenic conditions. The comparisons in panel A illustrate structural differences and do not represent biological conversion reactions. Abbreviations: AOB, ammonia-oxidizing bacteria; E1, estrone; E2, 17β-estradiol; E3, estriol.

**Figure 3:**
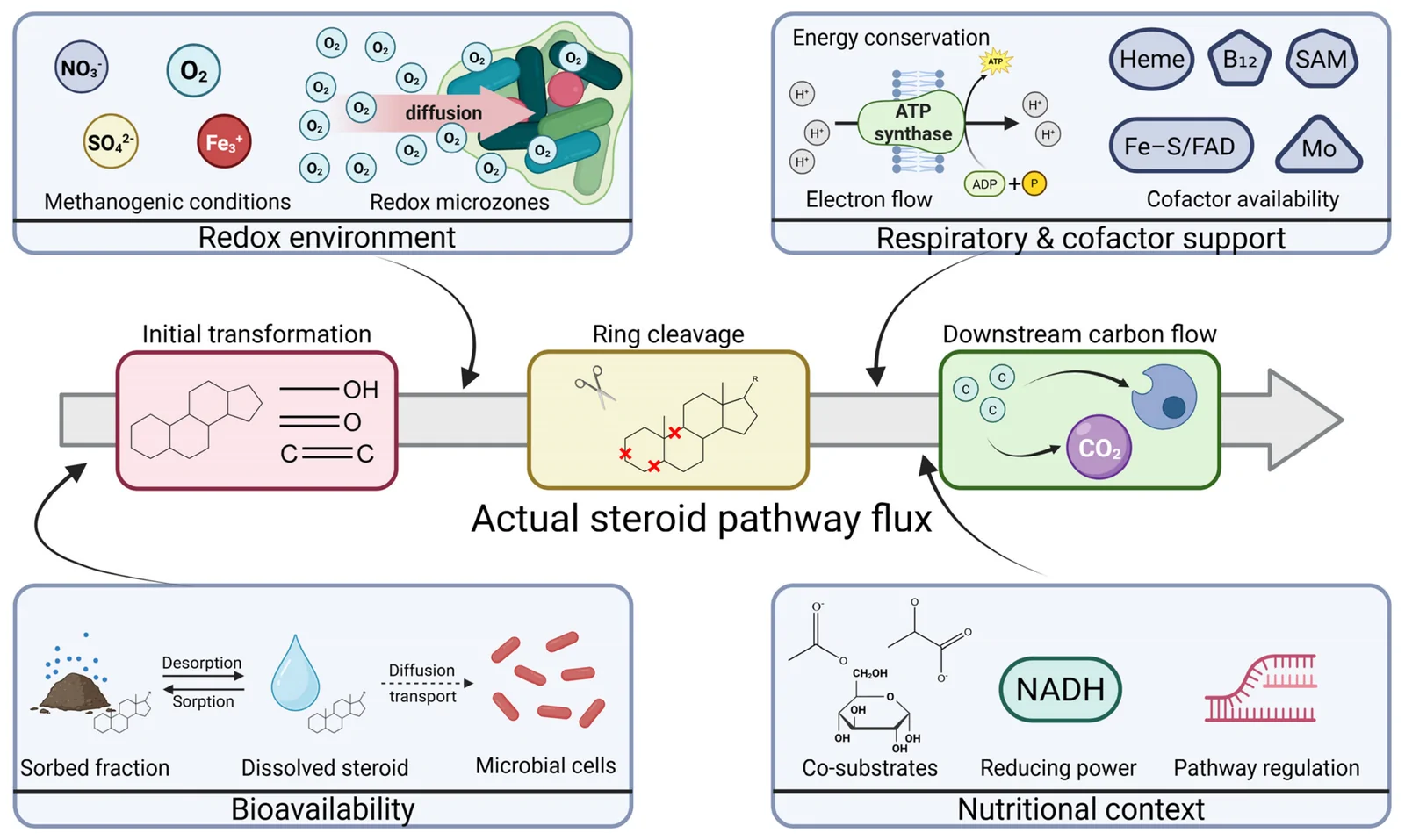
Environmental and physiological controls on microbial steroid transformation and downstream carbon flow.

**Figure 4:**
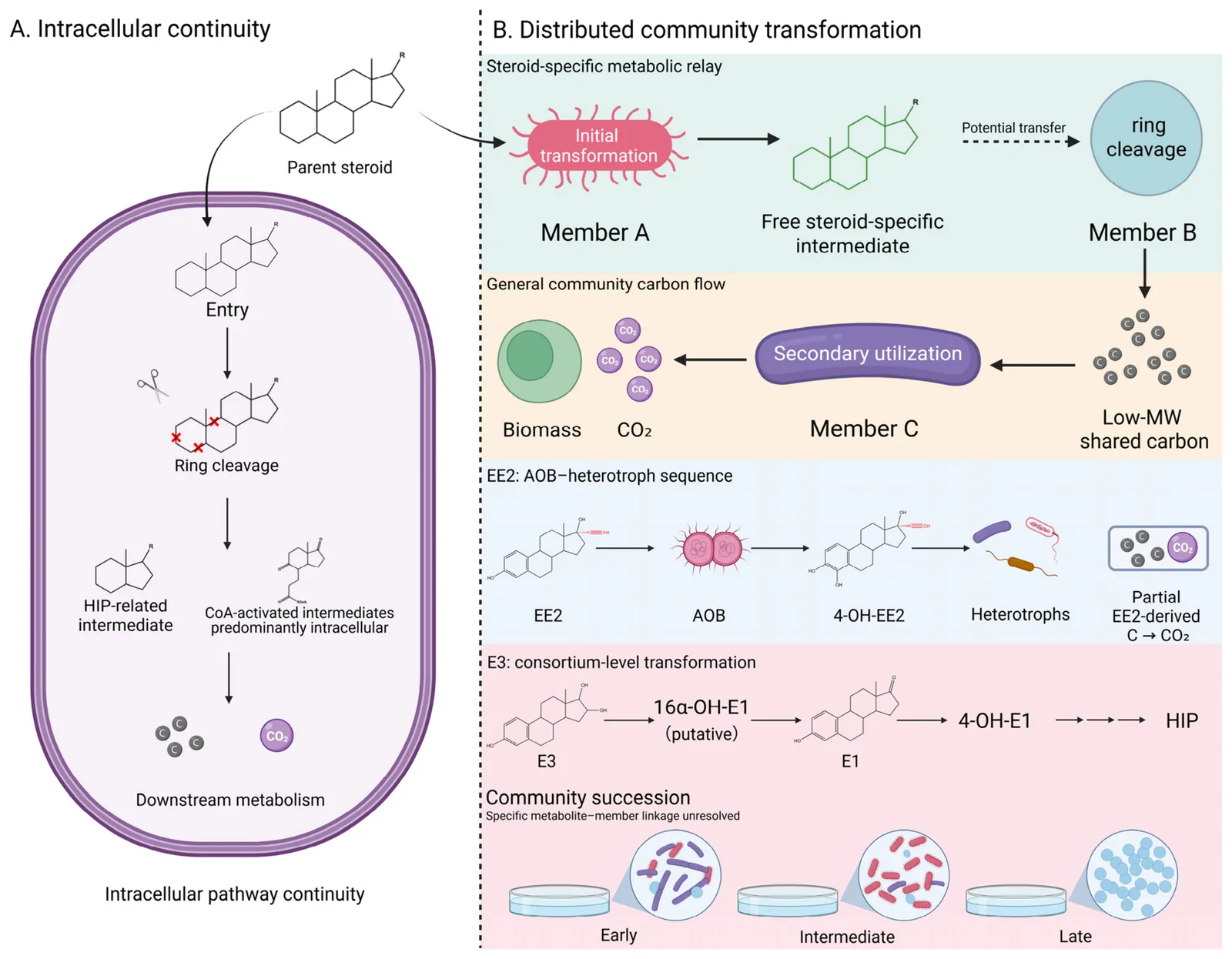
Intracellular pathway continuity and distributed community organization during microbial steroid transformation. (**A**) Relatively continuous steroid catabolism may connect initial transformation, steroid-ring cleavage, HIP-related processing, and downstream metabolism within the same functional member. CoA-activated downstream intermediates are predominantly intracellular and are therefore less likely to function as direct exchange metabolites between community members. (**B**) In distributed communities, free steroid-specific intermediates may connect functionally distinct members, whereas low-molecular-weight products generated after extensive steroid processing can enter general community carbon flow and support secondary utilization, biomass formation, and CO_2_ production. The EE2 ammonia-oxidizer–heterotroph sequence illustrates sequential transformation of EE2 through 4-hydroxy-EE2 followed by heterotrophic processing and partial conversion of EE2-derived carbon to CO_2_. The E3-enriched consortium illustrates sequential formation of putative 16α-hydroxyestrone, E1, 4-hydroxyestrone, 4,5-seco-related intermediates, and HIP together with temporal community succession; however, specific metabolite–member producer–receiver linkages remain unresolved. Abbreviations: AOB, ammonia-oxidizing bacteria; EE2, 17α-ethinylestradiol; E3, estriol; E1, estrone; HIP, 3aα-H-4α(3′-propanoate)-7aβ-methylhexahydro-1,5-indanedione; Low-MW, low molecular weight.

**Table 1:** Major microbial transformation and catabolic routes characterized for natural steroids.

Steroid/Condition	Representative System	Major Route	Key Mechanistic Features	Representative References
Sterols and androgens, aerobic	*Rhodococcus* and *Mycobacterium* spp.; *Comamonas testosteroni*	9,10-seco	For sterols, side-chain processing precedes C19 steroid-nucleus degradation, whereas androgens can enter the 9,10-seco module directly; KstD/KshAB mediate key activation steps in actinobacterial models.	[27,31,32,42]
Progesterone, aerobic	*Caenibius tardaugens*	C21→C19 remodeling → 9,10-seco	*pdc*-encoded BVMO and LLM convert progesterone to testosterone acetate, channeling it into androgen-like downstream catabolism.	[25]
Estrogens, aerobic	*Sphingomonas*, *Novosphingobium* and *Rhodococcus* spp.	4,5-seco	E2→E1 oxidation, C4 hydroxylation and A-ring cleavage; downstream processing differs among bacterial lineages.	[24,28,43,44,45,46]
Androgens, nitrate-reducing	*Steroidobacter denitrificans*; *Sterolibacterium denitrificans*	2,3-seco	AtcABC mediates 1-testosterone hydration/dehydrogenation, whereas SRAH catalyzes C2–C3 hydrolysis of a cyclic β-diketone; together, they define key steps of oxygen-independent A-ring cleavage.	[33,47,48,49]
Estrogens, nitrate-reducing	*Denitratisoma* sp. strain *DHT3*; *Denitratisoma oestradiolicum*	Methylation → 2,3-seco	Cobalamin-dependent estrogen-to-androgen conversion links estrogens to the 2,3-seco pathway; SAM was identified as the methyl donor in the biochemically characterized *D. oestradiolicum* system.	[30,50]
Estrogens, other anaerobic conditions	Mixed anaerobic communities	Skeleton-retaining transformation	E2↔E1 redox conversion occurs under several electron-accepting conditions; 17α-E2 is formed under Fe(III)-reducing, sulfate-reducing and methanogenic conditions.	[34]

Note: HIP-related CoA-dependent C/D-ring catabolism is particularly well characterized in actinobacteria [29,51,52]. Abbreviations: BVMO, Baeyer–Villiger monooxygenase; E1, estrone; E2, 17β-estradiol; HIP, 3aα-H-4α(3′-propanoate)-7aβ-methylhexahydro-1,5-indanedione; LLM, luciferase-like monooxygenase; SAM, S-adenosyl-L-methionine.

**Table 2:** Representative microbial transformation systems for synthetic steroid hormones.

Steroid/System	Physiological Context	Main Observation	Transformation Depth/Downstream Fate	Representative References
EE2, nitrifying sludge/AOB	Nitrification-associated cometabolism	EE2 transformation is closely associated with active ammonia oxidation	Initial transformation demonstrated; complete downstream pathway unresolved	[37,92,93]
EE2, natural-estrogen-degrading isolates	Natural-estrogen-dependent cometabolism	EE2 is transformed during metabolism of E1, E2, or E3	Specific EE2-derived downstream metabolites and carbon fate remain unresolved	[35]
EE2, AOB–heterotroph system	Sequential functional-group transformation	AOBs transform EE2, while heterotrophs further process EE2 and AOB-derived metabolites	Partial mineralization of EE2-derived carbon demonstrated; downstream stability differs among EE2 products	[88]
EE2, *Pseudomonas citronellolis* SJTE-3	Heterotrophic transformation with auxiliary energy sources	Two EE2 intermediates and genes associated with EE2 transformation were identified; the strain also removes EE2 in environmental matrices	Initial molecular components characterized; continuous ring-cleavage and carbon-fate pathway unresolved	[96,97]
EE2, *Aeromonas salmonicida* MLN-TP7	Heterotrophic transformation	High parent removal and several candidate downstream products were reported	Candidate downstream products proposed; EE2-derived carbon continuity not established	[98]
EE2, anaerobic mixed communities	Tested anaerobic conditions	No detectable EE2 biotransformation under the tested long-term conditions	No downstream pathway demonstrated	[34]
Norgestrel, activated-sludge-derived bacteria	Aerobic heterotrophic transformation	Multiple skeleton-retaining transformation products were identified	Initial redox/dehydrogenation reactions established; subsequent ring cleavage and carbon fate unresolved	[91]

Note: The table summarizes representative systems with distinct transformation modes and is not intended as an exhaustive inventory of synthetic-steroid studies; broader compound-level kinetic studies are summarized in Table S1. Abbreviations: AOB, ammonia-oxidizing bacteria; E1, estrone; E2, 17β-estradiol; E3, estriol; EE2, 17α-ethinylestradiol.

**Table 3:** Complementary observation dimensions for interpreting microbial transformation of environmental steroids.

Observation Dimension	Main Question	Representative Evidence	Representative Steroid Examples	Interpretation Supported
Metabolic potential	What steroid-transforming capacity may be present?	Genomes/metagenomes, functional genes and pathway–gene clusters	9,10-seco-associated modules [31,32]; estrogen 4,5-seco gene clusters [24,28,43,44,45,46]; *emtABCD* and 2,3-seco-associated genes [30,33,47,48,49]	Candidate pathway capacity and environmental distribution
Active transformation	What reactions actually occur under the tested conditions?	Time-resolved metabolites, substrate-dependent expression, enzyme assays and genetic validation	Aerobic E1/E2 4,5-seco pathway in strain KC8 [28]; estrogen-to-androgen conversion and subsequent 2,3-seco metabolism in *Denitratisoma* [30]	Reaction sequence, pathway operation and catalytic involvement
Steroid-derived carbon flow	Where does steroid-derived carbon go?	Isotope tracing to biomass and/or inorganic carbon, supported by carbon balance where available	[4-^14^C]testosterone assimilation and CO_2_ formation [48]; partial mineralization of EE2-derived carbon after heterotrophic processing [88]	Steroid-carbon assimilation and/or mineralization; member-level attribution requires additional resolved tracing
Endocrine-risk outcome *	Does transformation alter net endocrine activity?	Parent/product profiling combined with receptor- or cell-based bioassays	Reduced estrogenic activity during E1 4,5-seco transformation [28]; E2 degradation by *Rhodococcus* spp. accompanied by marked loss of estrogenic activity [62]	Change in biological effect associated with transformation products and parent removal

* Endocrine-risk outcomes represent a parallel environmental endpoint rather than a higher level of carbon-flow evidence. These observation dimensions are complementary and should not be interpreted as an evidence-quality ranking. Abbreviations: E1, estrone; E2, 17β-estradiol; EE2, 17α-ethinylestradiol.

## Data Availability

No new data were created or analyzed in this study. Data sharing is not applicable to this article.

## Supplementary Materials

The following supporting information can be downloaded at https://www.mdpi.com/article/10.3390/microorganisms14102160/s1, Table S1: Experimental context and evidence for representative microbial steroid-transformation studies discussed in this review.
