# Supplementary material for: Beyond Parent-Compound Removal: Microbial Transformation Pathways, Physiological Constraints, and Evidence Integration for Environmental Steroid Hormones
Source: Microorganisms. 2026 Sep 24;14(10):2160. doi: 10.3390/microorganisms14102160 (PMC13707411; doi:10.3390/microorganisms14102160)
Supplement: Supplementary file 1 [file microorganisms-14-02160-s001.zip › microorganisms-4562193-supplementary.pdf]

Table S1. Experimental context and evidence for representative microbial steroid-transformation studies discussed in this review.

Steroid(s)	System/organism	Experimental context	Main transformation outcome	Analytical/mechanistic evidence	Interpretive boundary	Reference
**Natural steroids**						
E1/E2	*Sphingomonas* sp. strain KC8	E1 cell suspensions (~1 mM); purified OecC assays with 4-OH-E1; aerobic defined medium	E2 → E1 → 4-OH-E1 → C4–C5 cleavage; PEA formation	NMR; genome/transcriptome; recombinant/purified OecC; Fe analysis; $^{18}O_2$ tracing	PEA may form by abiotic recyclization of the unstable cleavage product; complete carbon allocation not established	[28]
E1	Activated sludge; *Novosphingobium* sp. SLCC	Activated-sludge enrichment; 3.7 nM E1 assay for 24 h; aerobic	PEA and downstream A/B-ring products detected; *Novosphingobium* enriched	UPLC-ESI-HRMS; ^{13}C-E1; metagenomics; qPCR; pure-culture validation	Labeled PEA supports 4,5-seco entry at environmentally relevant concentration, but not complete mineralization	[67]
Testosterone	*Sterolibacterium denitrificans* DSM 13999	1 mM testosterone + [4-^{14}C]testosterone; 10 mM nitrate; 60 h; anoxic	2,3-seco metabolism; steroid-derived ^{14}C recovered in biomass and CO_2	^{14}C tracing; ^{13}C metabolomics; HPLC; growth and nitrate measurements	Direct carbon-fate evidence in a defined pure culture; carbon recovery was incomplete; no community-level carbon-transfer information.	[77]
E1/E2	*Denitratisoma* sp. strain DHT3	Denitrifying pure culture; nitrate-reducing; cobalamin-dependent	Estrogens → C19 androgen-type intermediates → 2,3-SAOA → HIP	Time-resolved metabolomics; isotope experiments; transcriptomics; *emtA* disruption; growth	Continuous transformation demonstrated; closed quantitative estrogen-carbon balance not available	[30]

		estrogen remodeling				
E1/E2	*Denitratisoma oestradiolicum* cell extracts	50 µM E1/E2; ATP/SAM or SAM-regeneration substrates; Ti(III)-citrate; anaerobic assay	SAM-dependent estrogen methylation → 1-dehydrotestosterone and related androgen products	Quantitative UPLC; cell-free assays; methyl-donor testing; methyl-^{13}C tracing	Methyl-donor biochemistry was characterized mainly in *D. oestradiolicum* and should be distinguished from DHT3 genetic evidence	[86]
E2/EE2	Lake water–sediment anaerobic communities	~5 mg L^{-1}; nitrate-reducing, Fe(III)-reducing, sulfate-reducing, and methanogenic incubations; prolonged EE2 testing	E2 ↔ E1; 17α-E2 under selected conditions; no detectable EE2 biotransformation	HPLC; GC-MS/MS; electron-acceptor reduction; sterile controls	Natural-estrogen products largely retained the steroid nucleus; sterile EE2 loss included sediment binding; ring cleavage not established	[34]
E3	IF-consortium from domestic sewage	0.1 mM E3 growth assays; 1 mM E3 pathway assay, 14 d; wastewater/sludge microcosms; aerobic	E3 → putative 16α-OH-E1 → E1 → 4-OH-E1 → PEA → HIP; community succession	GC-MS; UPLC-ESI-HRMS; time-resolved metabolites; 16S rRNA profiling; abiotic controls	DMSO can support growth; no labeled-E3 carbon balance; specific metabolite–member linkage unresolved	[41]
Progesterone	*Caenibius tardaugens* NBRC 16725	M63 medium; 1.71 mM progesterone + cyclodextrin; enzyme assays ~0.5 mM; aerobic	C21 progesterone → testosterone acetate/related C19 steroid esters	RNA-seq; targeted mutants; crude-extract assays; HPLC-MS; BVMO/LLM analysis	Defines an entry mechanism for natural progesterone; direct extrapolation to synthetic progestins is not warranted	[25]

HIP/cholesterol-derived intermediates	*Rhodococcus jostii* RHA1; *Mycobacterium* spp.	Growth assays including 1 mM cholesterol; intracellular CoA-metabolite analysis; aerobic	HIP activation and sequential CoA-dependent C/D-ring processing	Gene deletion/complementation; CoA metabolomics; synthetic standards; enzyme characterization	Detailed module best characterized in actinobacteria; CoA intermediates are predominantly intracellular	[29]
**Synthetic steroids and comparative systems**						
EE2	Nitrifying activated sludge	~50 μg L^{-1} EE2; high-nitrification enrichment; up to 6 d	EE2 disappearance with formation of more hydrophilic products	[^{3}H]EE2; radiochromatography; HPLC; ammonium oxidation measurements	Products unidentified; association with nitrification does not demonstrate direct AMO catalysis or mineralization	[37]
EE2 + E1/E2/E3	Six natural-estrogen-degrading isolates	~0.5 μg L^{-1} E2 + 0.6 μg L^{-1} EE2 for 4 d; additional higher-concentration assays	EE2 transformed only during natural-estrogen metabolism	HPLC; LC-MS/MS; GC-MS/MS; ATP measurements; substrate-ratio experiments	No stable additional EE2 products detected; metabolite non-detection does not establish ring cleavage or mineralization	[35]
EE2	*Nitrosomonas europaea* (AOB); heterotrophic cultures; nitrifying sludge	Chemostats, 500 ng L^{-1} to 1 mg L^{-1}; ^{14}C-EE2 experiments; sequential AOB→heterotroph reactors	AOB form 4-OH-EE2, sulfo-EE2 and other products; heterotrophs further transform products and generate $^{14}CO_2$	^{14}C mass balance; CO_2 trapping; LC-MS/LC-ITMS; NMR; ATU inhibition	Distinct AOB/heterotroph roles; sulfo-EE2 can persist; some nitro products may form abiotically at high nitrite	[88]
EE2	*Pseudomonas citronellolis* SJTE-3	Laboratory assays ~1–10 mg L^{-1}; environmental water, soil and manure	SDR3, YjcH, and CYP2 associated with EE2 transformation; substantial parent	HPLC/GC-MS; growth/CFU; functional analyses; environmental microcosms; boiled-cell controls	Ethanol/Tween 80 may affect energy supply and mass transfer; EE2-carbon assimilation/mineralization not established	[96,97]

		microcosms; aerobic	removal in environmental matrices			
EE2	*Aeromonas salmonicida* MLN-TP7	Mineral medium; 9 mg L^{-1} EE2; ~7–9 d; aerobic	High parent removal; E1 and lactone-, organic-acid- and TCA-like candidate products	GC-MS metabolite profiling; EE2 quantification; culture monitoring	Appreciable abiotic loss; no clear OD increase; possible methanol contribution; TCA-cycle entry/mineralization is not supported by isotope continuity	[98]
Norgestrel	Activated-sludge-derived bacterial isolates	Aerobic isolate assays; comparison with progesterone	4,5-Dihydro-, 3α,5β-tetrahydro- and 6,7-dehydro-norgestrel; one unidentified product	Parent/product analysis; isolate characterization	Reactions largely retain the steroid nucleus; substituent fate, ring cleavage and carbon fate unresolved	[91]
29 natural and synthetic steroids	Fresh municipal activated sludge	500 µg L^{-1} each; 28 °C; aerobic; up to 60 h	Compound-dependent apparent half-lives across steroid classes	UPLC-MS/MS; time-series kinetics; sterile controls	Useful for comparative persistence; parent-loss kinetics do not resolve entry reactions, ring cleavage or final carbon fate	[8]

Notes: Experimental conditions shown are those most directly relevant to the interpretations discussed in this review; individual studies may contain additional assays performed under other conditions. Parent-compound removal is distinguished from structural biotransformation, steroid-ring cleavage, and mineralization. Detection of central-metabolism-like products without isotope continuity is not considered direct evidence that the corresponding carbon originated from the steroid substrate.

Abbreviations: E1, estrone; E2, 17β-estradiol; E3, estriol; EE2, 17α-ethinylestradiol; 4-OH-E1, 4-hydroxyestrone; 16α-OH-E1, 16α-hydroxyestrone; PEA, pyridinestrone acid; 2,3-SAOA, 17β-hydroxy-1-oxo-2,3-seco-androstan-3-oic acid; HIP, 3aα-H-4α(3′-propanoate)-7aβ-methylhexahydro-1,5-indanedione; OecC, 4-hydroxyestrone 4,5-dioxygenase; AOB, ammonia-oxidizing bacteria; AMO, ammonia monooxygenase; ATU, allylthiourea; BVMO, Baeyer–Villiger monooxygenase; LLM, luciferase-like monooxygenase; SDR3, short-chain dehydrogenase 3; YjcH, YjcH-family transporter; CYP2, cytochrome P450 enzyme 2; SAM, S-adenosyl-L-methionine; ATP, adenosine triphosphate; CoA, coenzyme A; DMSO, dimethyl sulfoxide; CFU, colony-forming units; OD, optical density; TCA, tricarboxylic acid; NMR, nuclear magnetic resonance spectroscopy; HPLC, high-performance liquid chromatography; UPLC, ultra-performance liquid chromatography; LC-MS, liquid chromatography–mass spectrometry; LC-MS/MS, liquid chromatography–tandem

mass spectrometry; HPLC-MS, high-performance liquid chromatography–mass spectrometry; GC-MS, gas chromatography–mass spectrometry; GC-MS/MS, gas chromatography–tandem mass spectrometry; LC-ITMS, liquid chromatography–ion-trap mass spectrometry; UPLC-MS/MS, ultra-performance liquid chromatography–tandem mass spectrometry; UPLC-ESI-HRMS, ultra-performance liquid chromatography–electrospray ionization–high-resolution mass spectrometry; qPCR, quantitative polymerase chain reaction; RNA-seq, RNA sequencing; 16S rRNA, 16S ribosomal RNA.
